# Predicting streamflow in Peninsular Malaysia using support vector machine and deep learning algorithms

**DOI:** 10.1038/s41598-022-07693-4

**Published:** 2022-03-10

**Authors:** Yusuf Essam, Yuk Feng Huang, Jing Lin Ng, Ahmed H. Birima, Ali Najah Ahmed, Ahmed El-Shafie

**Affiliations:** 1 Department of Civil Engineering, Lee Kong Chian Faculty of Engineering and Science, Universiti Tunku Abdul Rahman, Selangor, Malaysia; 2 Department of Civil Engineering, Faculty of Engineering, Technology, and Built Environment, UCSI University, 56000 Kuala Lumpur, Malaysia; 3 Department of Civil Engineering, College of Engineering, Qassim University, Unaizah, Saudi Arabia; 4 Institute of Energy Infrastructure (IEI), Department of Civil Engineering, College of Engineering, Universiti Tenaga Nasional, 43000 Kajang, Selangor Malaysia; 5 Department of Civil Engineering, Faculty of Engineering, University of Malaya, Kuala Lumpur, Malaysia

**Keywords:** Hydrology, Engineering

## Abstract

Floods and droughts are environmental phenomena that occur in Peninsular Malaysia due to extreme values of streamflow (SF). Due to this, the study of SF prediction is highly significant for the purpose of municipal and environmental damage mitigation. In the present study, machine learning (ML) models based on the support vector machine (SVM), artificial neural network (ANN), and long short-term memory (LSTM), are tested and developed to predict SF for 11 different rivers throughout Peninsular Malaysia. SF data sets for the rivers were collected from the Malaysian Department of Irrigation and Drainage. The main objective of the present study is to propose a universal model that is most capable of predicting SFs for rivers within Peninsular Malaysia. Based on the findings, the ANN3 model which was developed using the ANN algorithm and input scenario 3 (inputs consisting of previous 3 days SF) is deduced as the best overall ML model for SF prediction as it outperformed all the other models in 4 out of 11 of the tested data sets; and obtained among the highest average RMs with a score of 3.27, hence indicating that the model is very adaptable and reliable in accurately predicting SF based on different data sets and river case studies. Therefore, the ANN3 model is proposed as a universal model for SF prediction within Peninsular Malaysia.

## Introduction

Floods and droughts are natural phenomena that have impacted regions within Peninsular Malaysia throughout recorded history. Recently, continuous heavy rainfall in January 2021 caused high streamflow (SF) within rivers and consequent widespread flooding in Peninsular Malaysia, with the state of Pahang representing the worst affected state. Approximately 50,000 individuals were evacuated, while at least six people died. Meanwhile, the worst water shortage affecting Peninsular Malaysia occurred back in 1998 when a prolonged drought caused very low amounts of SF and the drying up of dam reservoir water resources. Given the shortage, water was rationed for almost 150 days in the Klang Valley, affecting 3.2 million people. Ultimately, these phenomena can be understood to be a result of extreme values of SF^1^. Too high amounts of SF cause the stream to exceed its confinement and submerge surrounding land, causing floods. On the other hand, droughts are a result of too low amounts of SF which causes diminishing water resources as rivers and dam reservoirs dry up simultaneously. SF is even recognized by the World Meteorological Organization (WMO) as a significant predictor of droughts and has been used in existing studies to forecast drought indicators namely the standardized drought index (SDI) and standardized SF index (SSI)^2,3^. As history has shown, floods and droughts make the task of water resource management and allocation extremely difficult, while also affecting other industries and activities such as hydropower generation, agriculture, and environmental protection^1,4–6^. Additionally, existing studies have also demonstrated the correlation of SF with river suspended sediment load (SSL). SF data has been used to obtain better predictions of SSL^7–10^, hence highlighting the effects of SF on SSL, with higher amounts of SF typically causing higher SSLs. On top of that, streamflow also has an effect on the capacity of rivers to receive pollution. The water quality index (WQI) is commonly used to describe the water quality of streamflow and is affected by six substances namely biochemical oxygen demand, chemical oxygen demand, dissolved oxygen (DO), suspended solids, ammoniacal nitrogen, and potential for hydrogen^11^. Large streamflow are better in receiving and diluting away pollution discharge concentrated with these substances, while smaller streamflow are more easily polluted by these substances as they are not able to degrade the pollution discharge swiftly. Given the aforementioned factors, a means to predict SF is greatly significant, especially approaching or during periods of floods and droughts, particularly for municipal and environmental damage mitigation; water resource management; continuation of hydropower generation and agricultural activities; and SSL and WQI monitoring.

Machine learning (ML), a branch of artificial intelligence, has been studied and utilized for the purpose of SF prediction. ML algorithms are able to identify trends and patterns in a large database easily and continually improve in predictive ability with time, while not requiring much human intervention as they self-learn. For these reasons, ML is a valuable tool for modelling and predicting SF as different rivers have different SF magnitudes and behaviours, depending on the spatial and temporal variability as well as the water balance component heterogeneity of a particular river^1,12^. Existing studies from recent years have established and shown several ML algorithms capable of producing SF predictions of high accuracy while outperforming other ML algorithms, namely the support vector machine (SVM) and two deep learning algorithms: artificial neural network (ANN) and long short-term memory (LSTM). Standalone SVMs have been demonstrated to produce more accurate SF predictions compared to extreme learning machine (ELM), adaptive neuro-fuzzy inference system (ANFIS), multivariate adaptive regression splines (MARS), M5 model tree, and ANN^6,13–17^. Hybridization has also been studied to enhance the predictive ability of SVM for different case studies. Malik et al.^18^ hybridized SVM with ant lion optimization (ALO), multi-verse optimizer (MVO), spotted hyena optimizer (SHO), Harris’ hawks optimization (HHO), particle swarm optimization (PSO), and Bayesian optimization (BO), to predict the daily SF in the watershed of Naula, India. It was found that SVM hybridized with HHO (SVM-HHO) was superior in SF predictive performance compared to the other hybridized algorithms. The study by Tikhamarine, Souag-Gamane, and Kisi^1^ hybridized SVM with the grey-wolf optimizer (GWO), shuffled complex evolution (SCE) algorithm, MVO, and PSO, to predict SF for the Ain Bedra and Fermatou stations in Algeria, in which it was found that SVM hybridized with GWO (SVM-GWO) outperformed the other hybridized algorithms. One of the primary advantages of the SVM that makes it perform well in SF prediction is that it is able to deal with overlearning and high dimensionality which may cause computational complexity and local extremum^19^. In addition, tuning or adjustment of only a few hyperparameters need to be performed, hence giving SVM a simple structure and ease of implementation^20,21^. However, the SVM’s predictive ability is negatively affected when the utilized data set is significantly noisy, as SVMs are sensitive to noise^22–24^. Meanwhile, standalone ANNs have been shown to produce superior performances in SF prediction compared to linear regression (LR), autoregressive integrated moving average (ARIMA), genetic expression programming (GEP), ANFIS, and SVM^25–29^. The studies by Zaini et al.^30^ and Sammen et al.^31^ on Malaysian rivers demonstrated improved ANN predictive performance when hybridized with the bat algorithm (BA) and sunflower optimization algorithm (SFA). ANN hybridization was also performed in the study by Li et al.^32^ using empirical mode decomposition (EMD), ensemble empirical mode decomposition (EEMD), and discrete wavelet transformation (DWT). It was found that ANN hybridized with EEMD (EEMD-ANN) was the best performing model in the respective study. In addition, the predictive performance of ANN was shown to be improved through the utilization and integration of additional data mining techniques, as shown by Zamanisabzi et al.^33^ in the study on the Elephant Butte Reservoir. SF was also able to be predicted accurately through the modelling of the relationship between SF and rainfall, as proven in the study by Ali and Shahbaz^34^ on Pakistan rivers. The upsides that make ANN powerful in SF prediction include being able to easily handle large data sets; detect complex non-linear relationships between input and output parameters; and relate input and output parameters without the utilization of complex mathematical models or calculations^35–37^. A drawback of the ANN is that it is computationally expensive and has a high dependence on the capability of available hardware^38–40^. This means that adequate processing power is required for models to be trained with realistic and efficient training durations. Apart from ANN, LSTM is another deep learning algorithm that has produced good performances in SF prediction. Standalone LSTMs have outperformed other algorithms such as the nonlinear autoregressive exogenous model (NARX), Gaussian process regression (GPR), SVM, ANN, and the standard technique of hydrological model parameters regionalization also known as the HMREG scheme^41–43^. LSTMs have also been hybridized to improve their performances in SF prediction for different case studies. The study by Ghimire et al.^44^ on the Brisbane River and Teewah Creek in Australia hybridized LSTM with the convolutional neural network (CNN), resulting in SF predictive performances outperforming algorithms such as gradient boosting regression (GBM), extreme gradient boosting (XGB), decision tree (DT), ELM, and MARS. Liu et al. developed an algorithm hybridizing Encoder Decoder LSTM with EMD, which was capable of producing accurate SF predictions for the case study of the Yangtze River, China^45^. The advantages of the LSTM which are the strong abilities to capture long-term time dependencies between input and output parameters; and to learn relationships within complex and high-dimensional data sets, contributes to its good performances in the field of SF prediction^46,47^. The downside of the LSTM is that it also requires high computational power to train and develop models in a reasonable timeframe, given that it is a deep learning ML algorithm^48,49^. An LSTM model may also take a longer time to train and develop depending on the difficulty of the problem to be solved as well as the LSTM architecture chosen^50^. Additionally, the LSTM is also prone to overfitting effects^51,52^, which may be reduced with the help of dropout regularization and early call-back mechanisms. Apart from these established algorithms (SVM, ANN, LSTM), other ML algorithms with good potential that have been developed and focused on for the purpose of accurate SF prediction include variations of ELM, ANFIS, and random forest (RF)^4,5,15,45,53,54^.

Based on the aforementioned existing studies, it can be found that majority have developed SF prediction models based on data from only one hydrological station or river. As SF is affected by factors namely spatial variability, temporal variability, and water balance component heterogeneity, the magnitude and behaviour of SF in different rivers often vary^1,12^. Due to this, the suitability of ML algorithms for SF prediction may also vary based on different rivers. Certain ML models or algorithms may excel in predicting SF accurately for a particular river but perform poorly in predicting SF for a different river, as they may be unable to effectively capture the behaviour of SF for the different river. Existing studies in Peninsular Malaysia have developed ML algorithms namely LR, M5P tree, RF, SVM, ANFIS, ARIMA, ANN, and LSTM to predict SF in rivers such as Sungai Muda in Kedah; Sungai Kuantan and Sungai Kenau in Pahang; Sungai Kelantan in Kelantan; and Sungai Kurau, Sungai Bernam, and Sungai Tualang in Perak^26,29–31,42,53^. Aside from the studies by Zaini et al.^30^, Sammen et al.^31^, and Pandhiani et al.^53^ which utilized data sets from two hydrological stations or rivers to develop SF prediction models, other SF prediction studies in Peninsular Malaysia have focused on data sets from only one hydrological station or river. This brings up a research gap in which it is unknown whether there exists a single ML model or algorithm that has the ability of accurately predicting SF for the many different rivers within Peninsular Malaysia, as there are no existing studies that have developed and tested ML models or algorithms based on data sets from a substantial number of rivers within the region. Therefore, the present study intends to undertake this research gap by developing SF prediction models based on SF time series data sets of hydrological stations located along 11 different rivers throughout Peninsular Malaysia. The ML algorithms utilized for SF prediction in the present study are the SVM, ANN, and LSTM. This is because the conducted literature review has shown them to produce accurate SF predictions as well as outperforming other ML algorithms in the field of SF prediction, hence indicating their superiority in this field. Additionally, the literature review performed has highlighted the algorithms’ noteworthy advantages which make them suitable to be used for SF prediction in the present study. Hybridization of SVM, ANN, and LSTM is not investigated in the present study, as the present study intends to identify the standalone ML model that is most accurate and suitable as a universal model for the case study of 11 different river streamflow data sets in Peninsular Malaysia, which has not been performed before in existing studies. The findings of the present study may then open up a topic or focus for a future study on the hybridization of the standalone universal model proposed at the end of the present study.

Real-life adoption and application of an ML model proposed from scientific literature for the purpose of SF prediction may be complicated due to doubt on whether the proposed ML model is able to reproduce its accurate performance for different river case studies, which may have different SF magnitudes and behaviours due to variability on a spatial and temporal scale, as well as varying heterogeneity in water balance components. Meanwhile, the development of individual or personalized SF predictive ML models for each river within a region is resource intensive as it may require a significant amount of time and cost. Rather than using up lots of resources to develop many tailor-made SF predictive ML models for each river within a region, it would be more resource-friendly to identify one ML model that is capable of predicting SF with good accuracy for many different rivers within a region. Therefore, the present study was motivated by the idea of proposing a single universal ML model that has been substantially and simultaneously tested on different rivers; and is capable of accurately predicting SF for any river case study within Peninsular Malaysia. The main contribution of the present study is the testing and development of SF prediction models using 3 ML algorithms and SF data sets of hydrological stations from 11 different rivers throughout Peninsular Malaysia; and the proposal of the best performing ML model in the present study as the universal model for accurate SF prediction in the region. The best performing ML model is selected by considering two factors, which are the number of times a model produced the most accurate predictive performance for a data set, and the reliability of each model in producing relatively high-accuracy predictions for the different data sets. The accuracy of the ML models in the present study is quantified through the utilization of selected performance evaluation measures, namely mean absolute error (MAE), root mean squared error (RMSE) coefficient of determination (R^2^) and ranking mean (RM). The findings from the present study may interest hydrological authorities or institutions that are searching for substantially tested ML models within Peninsular Malaysia, or even other regions. The rest of the present study is organized as follows: “Materials and methods” describes the materials and methods used to develop and test the SF prediction models. Section “Results and discussion” reports and discusses the performance of the SF prediction models. Section “Conclusion” concludes the overall study and provides suggestions for future studies.

## Materials and methods

The materials and methods used in the developing and testing of SF predictions models for the 11 selected rivers within Peninsular Malaysia are explained in this section. Information on the location and data of case study, model development process, feature selection; data pre-processing; ML algorithms; and performance measures are described.

### Location and data of case study

The western region of Malaysia is known as Peninsular Malaysia. It comprises of 13 states and 2 federal territories; and has an area of approximately 132,265 km^2^. Located just North of the equator, Peninsular Malaysia consists of 40% of Malaysian land. Malaysia’s capital is the Federal Territory of Kuala Lumpur, which is located about 40 km from the coast. There are approximately 1235 river basins in Peninsular Malaysia, of which 74 are classified as main river basins while the remaining 1161 are categorized as small river basins^55^. The longest river in Peninsular Malaysia is Sungai Pahang, measuring up to 459 km in length.

The raw daily average SF data for different rivers within 11 states in Peninsular Malaysia was obtained from the Water Resources Management and Hydrology Division of the Malaysian Department of Irrigation and Drainage. To conduct the present study, one river is selected per state based on suitability of data in terms of volume and time series continuity; and the significance of the river to their respective state or federal territory. Table 1 provides information on the selected rivers for each state, the SF station numbers as well as latitudes and longitudes, and the data duration provided by each SF station.

**Table 1:** Information on selected rivers’ data for each state.

State	River	SF station no.	Latitude	Longitude	Data duration
Johor	Sungai Johor	1,737,451	01°46′50″N	103°44′45″E	1978 to 1998
Kedah	Sungai Muda	5,605,410	05°36′35″N	100°37′35″E	1976 to 2009
Kelantan	Sungai Kelantan	5,721,442	05°45′45″N	102°09′00″E	1980 to 1997
Melaka	Sungai Melaka	2,322,413	02°20′35″N	102°15′10″E	1979 to 2004
Negeri Sembilan	Sungai Kepis	2,723,401	02°42′20″N	102°21′20″E	1980 to 1995
Pahang	Sungai Pahang	3,527,410	03°30′45″N	102°45′30″E	1988 to 2009
Perak	Sungai Perak	4,809,443	04°49′10″N	100°57′55″E	1977 to 1995
Perlis	Sungai Arau	6,503,401	06°30′10″N	100°21′05″E	1986 to 1995
Selangor	Sungai Selangor	3,414,421	03°24′10″N	101°26′35″E	1976 to 2001
Terengganu	Sungai Dungun	4,832,441	04°50′35″N	103°12′15″E	1977 to 1996
F.T. of Kuala Lumpur	Sungai Klang	3,116,430	03°08′20″N	101°41′50″E	2010

### Model development process

**Figure 1:**
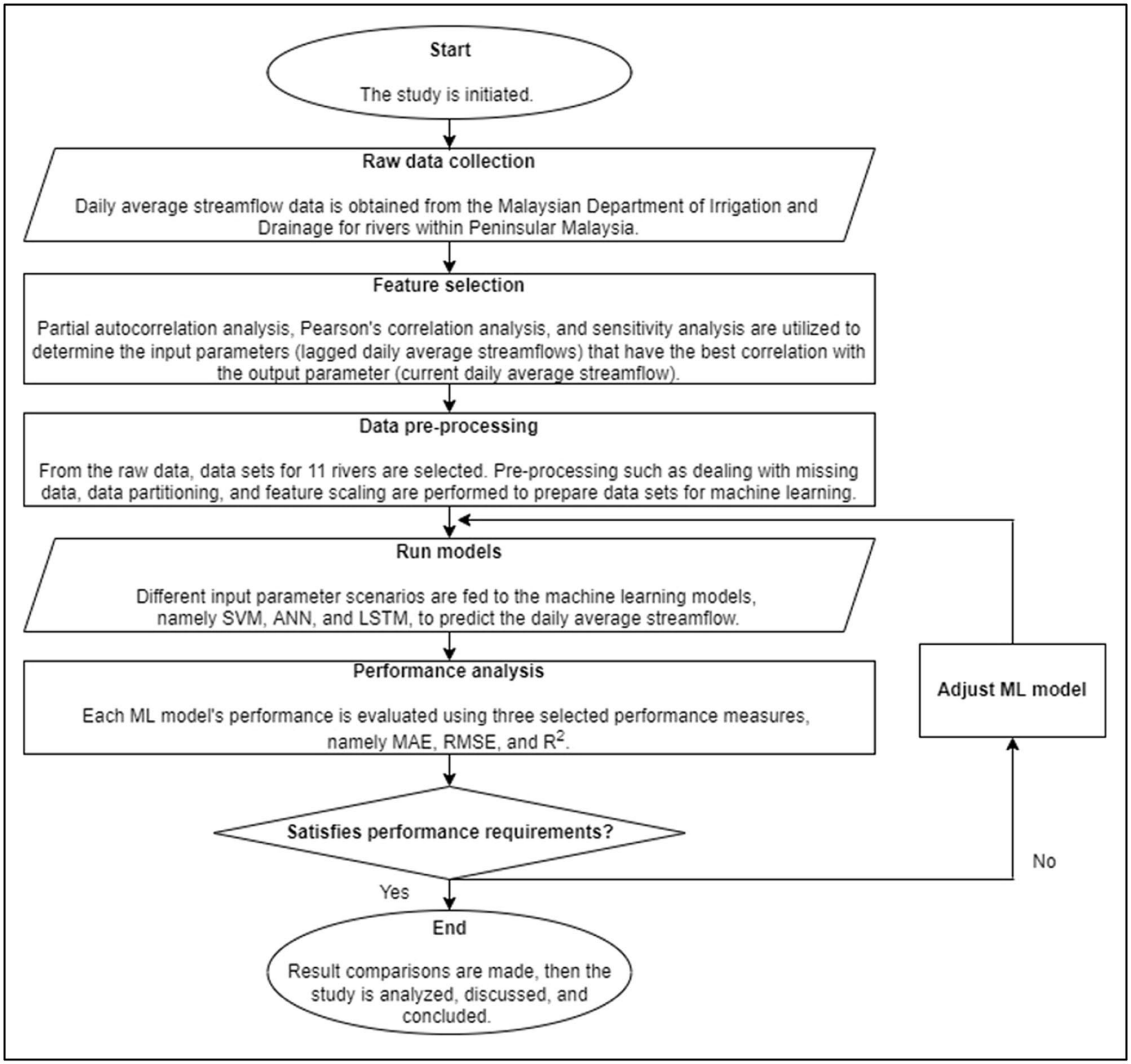
The SF prediction model development process employed.

The processes used to develop and test the SF prediction models in the present study comprises of raw data collection, feature selection, data pre-processing, model prediction, and performance analysis. The model development process employed in the present study is illustrated in Fig. 1.

### Feature selection

The process of selecting input parameters to be fed to an algorithm for model training is known as feature selection. It is important as a means to identify input parameter combinations that would enable accurate model predictions. For the present study, only the daily average streamflow (SF) data was available and utilized to predict future SF, hence the present study is categorized as univariate. A statistical analysis on the daily average SF for each of the 11 selected rivers is shown in Table 2.

**Table 2:** Statistical analysis of SF data for the 11 selected rivers.

River data set	Mean (m^3^/s)	Median (m^3^/s)	Mode (m^3^/s)	Std. dev. (m^3^/s)	Min. (m^3^/s)	Max. (m^3^/s)	Count
Sungai Johor, Johor	40.0	28.7	15.9	43.4	0.5	709.7	7670
Sungai Muda, Kedah	87.1	58.0	26.0	88.3	3.0	1160.0	12,419
Sungai Kelantan, Kelantan	495.7	364.2	509.3	587.1	81.7	9775.1	6575
Sungai Melaka, Melaka	5.8	3.3	1.4	7.8	0.0	119.9	9497
Sungai Kepis, Negeri Sembilan	0.5	0.2	0.1	1.7	0.0	65.3	5844
Sungai Pahang, Pahang	683.5	520.6	497.3	540.5	133.0	6285.3	8036
Sungai Perak, Perak	219.6	212.0	250.0	109.7	19.0	988.0	6939
Sungai Arau, Perlis	0.7	0.0	0.0	1.6	0.0	23.0	3652
Sungai Selangor, Selangor	53.9	44.0	34.7	36.7	2.3	313.9	9497
Sungai Dungun, Terengganu	124.5	78.2	50.0	185.1	9.4	3178.8	7305
Sungai Klang, F.T. of Kuala Lumpur	0.5	19.7	20.8	9.6	10.3	105.6	365

**Figure 2:**
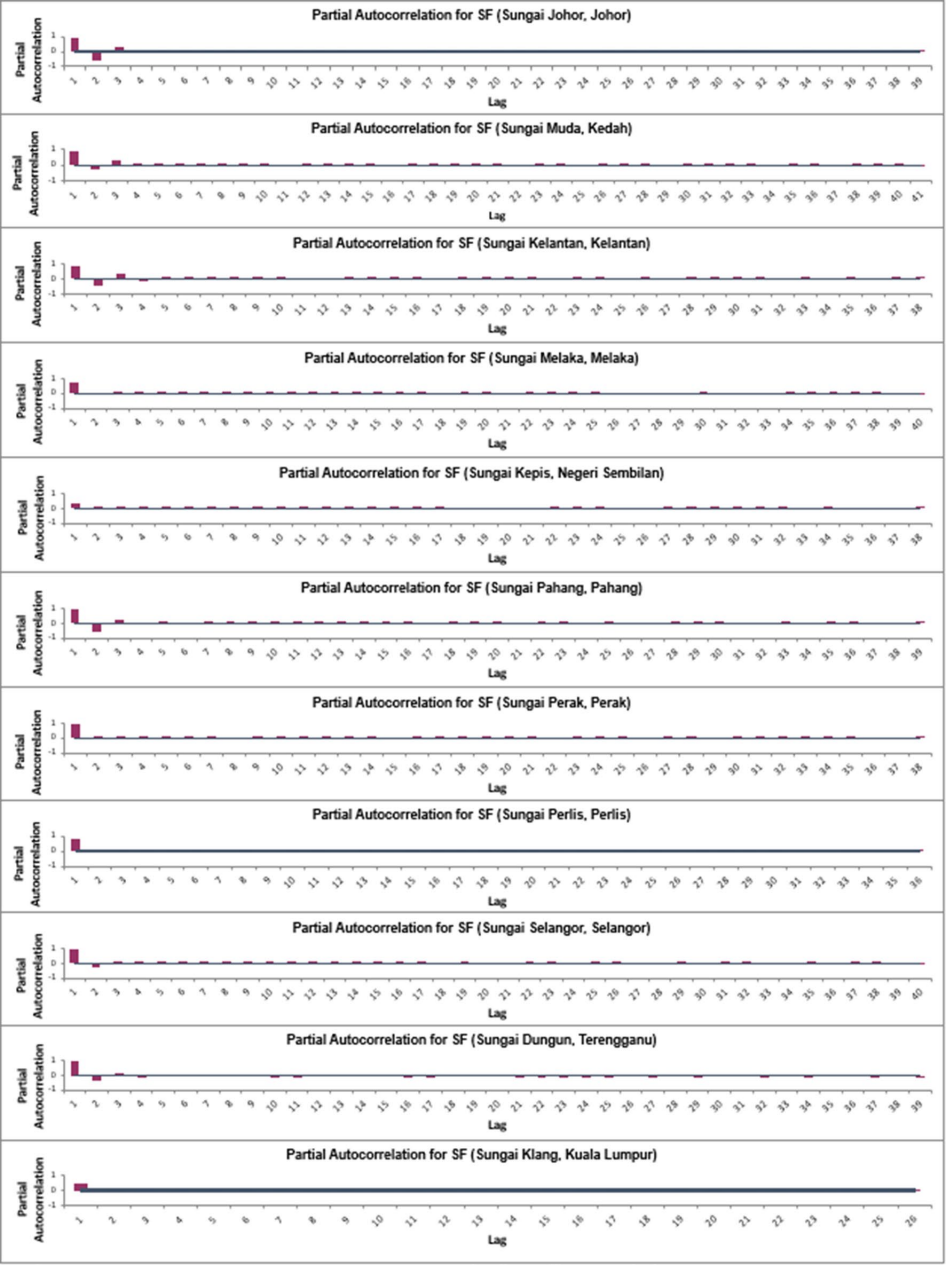
Partial autocorrelogram for SF for all data sets.

Given that the present study is univariate and two of the algorithms to be tested (SVM and ANN) are not traditional time-series forecasting algorithms, the SF data sets for each river are organized into sliding windows in order to reframe the time-series forecasting problem into a supervised learning problem. Before the data sets were organized into sliding windows, partial autocorrelation function (PACF) analyses were carried out on all the SF data sets in order to identify the lagged SF data that have significant correlation to the current-day SF data. Based on Fig. 2, it is found that for many of the SF data sets, the lagged SFs that are significantly correlated to the current-day SF [SF(t)] are the 1-day lagged SF [SF(t − 1)], 2-day lagged SF [SF(t − 2)], and 3-day lagged SF [SF(t − 3)].

In addition, the Pearson’s correlation coefficient is utilized to further analyse and understand the correlation between the current-day SF data [SF(t)] and the selected lagged SF data [SF(t − 1), SF(t − 2), SF(t − 3)]. The mathematical formula used to calculate Pearson’s correlation coefficient, symbolized by ${r}_{xy},$ is represented by:

1 $${r}_{xy}=\frac{\sum_{i=1}^{n}\left({x}_{i}-\overline{x }\right)\left({y}_{i}-\overline{y }\right)}{\sqrt{\sum_{i=1}^{n}{\left({x}_{i}-\overline{x }\right)}^{2}}\sqrt{\sum_{i=1}^{n}{\left({y}_{i}-\overline{y }\right)}^{2}}}$$

where $\overline{x }$,$\overline{y }$ are respective data means; ${x}_{i},{y}_{i}$ are individual respective data points; and $n$ is the sample size.

Through the calculation of Pearson’s correlation coefficient, it is found that there is indeed strong correlation between current-day SF data [SF(t)] and the selected lagged SFs [SF(t − 1), SF(t − 2), SF(t − 3)] in majority of the data sets. Table 3 shows Pearson’s correlation coefficient matrix for all 11 SF data sets used in the present study.

**Table 3:** Pearson’s correlation coefficient matrix for data sets of each selected river.

Pearson correlations	SF(t)	SF(t − 1)	SF(t − 2)	SF(t − 3)
**Pearson's correlation coefficient matrix based on Sungai Johor, Johor data set**				
SF(t)	1	0.9573	0.8632	0.7593
SF(t − 1)		1	0.9573	0.8632
SF(t − 2)			1	0.9573
SF(t − 3)				1
**Pearson's correlation coefficient matrix based on Sungai Muda, Kedah data set**				
SF(t)	1	0.9263	0.8143	0.7371
SF(t − 1)		1	0.9263	0.8143
SF(t − 2)			1	0.9263
SF(t − 3)				1
**Pearson's correlation coefficient matrix based on Sungai Kelantan, Kelantan data set**				
SF(t)	1	0.9035	0.7371	0.6133
SF(t − 1)		1	0.9035	0.7371
SF(t − 2)			1	0.9035
SF(t − 3)				1
**Pearson's correlation coefficient matrix based on Sungai Melaka, Melaka data set**				
SF(t)	1	0.7939	0.6104	0.5285
SF(t − 1)		1	0.7939	0.6104
SF(t − 2)			1	0.7939
SF(t − 3)				1
**Pearson's correlation coefficient matrix based on Sungai Kepis, Negeri Sembilan data set**				
SF(t)	1	0.3871	0.2223	0.1150
SF(t − 1)		1	0.3871	0.2224
SF(t − 2)			1	0.3871
SF(t − 3)				1
**Pearson's correlation coefficient matrix based on Sungai Pahang, Pahang data set**				
SF(t)	1	0.9744	0.9193	0.8579
SF(t − 1)		1	0.9744	0.9193
SF(t − 2)			1	0.9744
SF(t − 3)				1
**Pearson's correlation coefficient matrix based on Sungai Perak, Perak data set**				
SF(t)	1	0.9474	0.9060	0.8801
SF(t − 1)		1	0.9474	0.9060
SF(t − 2)			1	0.9475
SF(t − 3)				1
**Pearson's correlation coefficient matrix based on Sungai Perlis, Perlis data set**				
SF(t)	1	0.8345	0.6837	0.6070
SF(t − 1)		1	0.8345	0.6838
SF(t − 2)			1	0.8345
SF(t − 3)				1
**Pearson's correlation coefficient matrix based on Sungai Selangor, Selangor data set**				
SF(t)	1	0.9438	0.8604	0.7930
SF(t − 1)		1	0.9438	0.8604
SF(t − 2)			1	0.9438
SF(t − 3)				1
**Pearson's correlation coefficient matrix based on Sungai Dungun, Terengganu data set**				
SF(t)	1	0.9293	0.8097	0.7007
SF(t − 1)		1	0.9293	0.8097
SF(t − 2)			1	0.9293
SF(t − 3)				1
**Pearson's correlation coefficient matrix based on Sungai Klang, F.T. of Kuala Lumpur data set**				
SF(t)	1	0.4886	0.2987	0.2989
SF(t − 1)		1	0.4894	0.2993
SF(t − 2)			1	0.4888
SF(t − 3)				1

The PACF and Pearson’s correlation coefficient analyses show that the selected lagged SF data [SF(t − 1), SF(t − 2), SF(t − 3)] have strong predictive powers in predicting the current-day SF data [SF(t)], hence they are selected to be used as input parameters in the present study. Using these input parameters, three input parameter scenarios are designed and fed to the selected ML algorithms for model training. By feeding and testing different input parameter scenarios to the ML algorithms for model training as performed by existing studies^4,6,15,18,34,43,56^, the sensitivity of the models to different input combinations is able to be analysed and understood; and the best input parameter combination for accurate SF predictions can be determined. Table 4 describes the input parameter scenarios used in the present study. In total, 99 models were run and evaluated, given 3 input parameter scenarios, 3 ML algorithms, and 11 different SF data sets.

**Table 4:** Input parameter scenarios designed for the present study.

Output parameter	Input parameter scenario	Input parameter(s)	Description
SF(t)	1	SF(t − 1)	When SF data of previous day is available
	2	SF(t − 1) + SF(t − 2)	When SF data of previous 2 days is available
	3	SF(t − 1) + SF(t − 2) + SF(t − 3)	When SF data of previous 3 days is available

### Data pre-processing

This section explains the pre-processing steps performed on the raw SF time-series data sets of the 11 selected rivers obtained from the Malaysian Department of Irrigation and Drainage. The data pre-processing steps comprise of the imputation of missing data, data partitioning, and feature scaling.

#### Missing data

**Figure 3:**
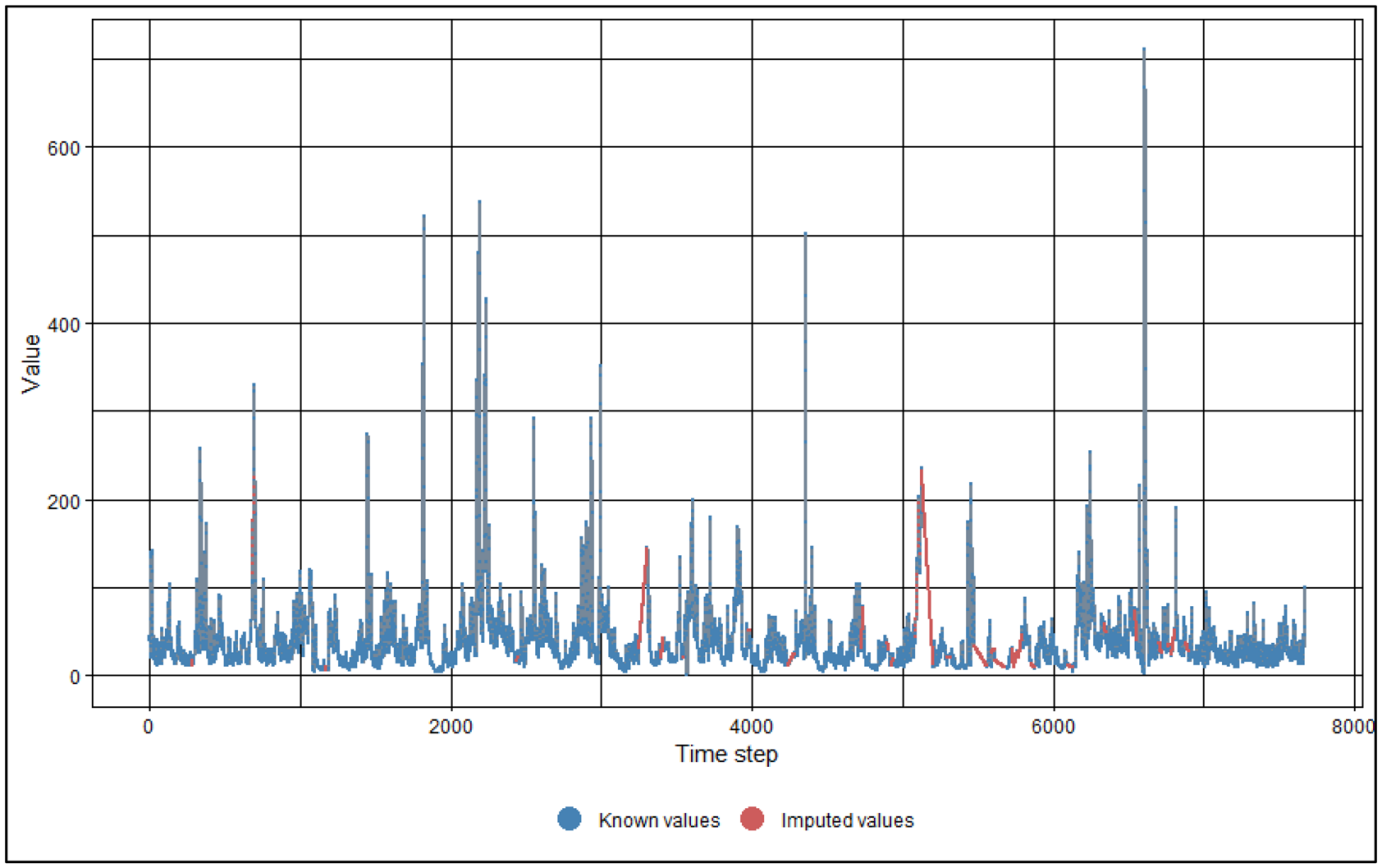
SF imputed values for Johor data set (SF values in units of m^3^/s, time step in units of day).

Machine learning algorithms generate errors when missing values are encountered within a data set. For this reason, the raw SF time-series data sets obtained from the Malaysian Department of Irrigation and Drainage needed to be processed as they contained missing SF values. In existing SF studies, missing data has been imputed by interpolation or filling in the measing values with mean or average; or by removing the missing data rows completely^12,26,27,54^. In the present study, imputation through interpolation is utilized to fill in the missing data. The imputation is carried out using the imputeTS R-package developed by Moritz and Bartz-Beielstein^57^. Linear interpolation and spline interpolation were tested to occupy the missing data sections. It was found that spline interpolation filled in some missing SF data with negative values, which is not logical as the water in the rivers move in only one direction. Therefore, linear interpolation was selected to inhabit the missing data portions. As a sample, the outcome of the imputation process for missing SF values in the Johor data set is shown in Fig. 3.

#### Data partitioning

The SF data sets in the present study are partitioned into two subsets, which are the training set and the test set. The training set is to be used for developing and providing the ML models with the ability to make SF predictions, while the test set is used for the evaluation of the ML models’ predictive ability using selected performance measures. An optimum ratio for the amount of training data to testing data is found to be 80:20, according to Kannangara et al.^58^. Existing SF prediction studies have also demonstrated good results using an 80:20 ratio for the amount of training data to testing data^6,26^. Therefore, 80% of each river’s SF data is used for training while the remaining 20% is used for testing in the present study. The training data is further split into a training set and a validation set. The validation set has the purpose of fine-tuning the model after each epoch, hence improving the model performance. The size of the validation set was selected through a trial-and-error process, in which it was found that using 20% of the training data as the validation set produced the best results for SF prediction. The duration of the training and testing set for each river after data partitioning can be seen in Table 5.

**Table 5:** Data partitioning for each river’s data set.

River data set	Total duration	Training set	Test set
Sungai Johor, Johor	1st January 1978 to 31st December 1998	1st January 1978 to 26th October 1994	27th October 1994 to 31st December 1998
Sungai Muda, Kedah	1st January 1976 to 31st December 2009	1st January 1976 to 22nd March 2003	23rd March 2003 to 31st December 2009
Sungai Kelantan, Kelantan	1st January 1980 to 31st December 1997	1st January 1980 to 2nd June 1994	3rd June 1994 to 31st December 1997
Sungai Melaka, Melaka	1st January 1979 to 31st December 2004	1st January 1979 to 27th October 1999	28th October 1999 to 31st December 2004
Sungai Kepis, Negeri Sembilan	1st January 1980 to 31st December 1995	1st January 1980 to 25th October 1992	26th October 1992 to 31st December 1995
Sungai Pahang, Pahang	1st January 1988 to 31st December 2009	1st January 1988 to 14th August 2005	15th August 2005 to 31st December 2009
Sungai Perak, Perak	1st January 1977 to 31st December 1995	1st January 1977 to 20th March 1992	21st March 1992 to 31st December 1995
Sungai Arau, Perlis	1st January 1986 to 31st December 1995	1st January 1986 to 7th January 1994	8th January 1994 to 31st December 1995
Sungai Selangor, Selangor	1st January 1976 to 31st December 2001	1st January 1976 to 26th October 1996	27th October 1996 to 31st December 2001
Sungai Dungun, Terengganu	1st January 1977 to 31st December 1996	1st January 1977 to 7th January 1993	8th January 1993 to 31st December 1996
Sungai Klang, F.T. of Kuala Lumpur	1st January 2010 to 31st December 2010	1st January 2010 to 26th October 2010	27th October 2010 to 31st December 2010

#### Feature scaling

As SVM and the deep learning algorithms (ANN and LSTM) are sensitive to data scales, feature scaling needs to be carried out on the SF data sets of each river. Feature scaling ensures that data variables are weighted accurately, so that convergence is fast and errors are minimized during training^43^. Depending on the ML algorithm to be used, two types of feature scaling methods are utilized, namely normalization and standardization. The present study utilizes standardization before training the SVM models, and normalization before training the deep learning models. Feature scaling is performed on the input data, which is determined through feature selection processes to be the 1-day, 2-day, and 3-day lagged SF; and the output data, which is the current-day SF. The outputs or raw predictions from the ML models are then inverse transformed back into their original scales in order to correctly proceed with evaluation and comparison through the usage of selected performance measures.

### Machine learning algorithms

In the present study, established ML algorithms in the field namely SVM and two deep learning algorithms: ANN and LSTM, were selected for development and testing of SF prediction models. SVM, ANN, and LSTM are regarded as established in the field of SF prediction due to the numerous studies demonstrating their effectiveness in recent years^1,6,13–18,25–34,41–44,59^. The Python programming language was utilized in the development and testing of the SF prediction models due to ease in commanding and comprehending the language, as well as its vast library support. Table 6 details the experimental setup used in developing the SF prediction models.

**Table 6:** Experimental setup.

Experimental setup parameter	Specification
Programming language	Python 3.7.12
ML libraries	Scikit-learn 1.0.1 (for SVM) TensorFlow 2.7.0 (for ANN, LSTM)
Notebook environment	Jupyter (hosted by Colaboratory)
Central processing unit (CPU)	Intel® Core™ i7-6700HQ CPU @ 2.60 GHz
Random access memory (RAM)	16.0 GB
System type	64-bit operating system, × 64 based processor

#### Support vector machine (SVM)

The SVM is a kernel-based algorithm that utilizes structural risk reduction and statistical learning methods in order to produce a good generalization capacity through the minimization of generalization error in contrast to training error^1,13,17^. SVM works by using a transfer function to non-linearly map input vectors into a high dimensional feature space, which helps to reduce the complexity of optimization^13,17^. The inspiration behind the SVR technique is the definition of a regression function approximation based on a set of support vectors originating from a training data set^1^. According to existing studies^1,17^, the SVM function is given by:

2 $$f\left(x\right)=\sum_{i=1}^{N}{(\alpha }_{i}-{\alpha }_{i}^{*})K(x,z)+{b}_{i}$$

where ${(\alpha }_{i}-{\alpha }_{i}^{*})$ is the Lagrange multiplier, $K(x,z)$ is the kernel function inside the multiplier, and ${b}_{i}$ is bias.

The kernel function represents the main SVR hyperparameter that requires to be selected or tuned before running the SVR models. The kernel functions that can be employed are the radial basis function (RBF), linear, polynomial, and sigmoid. Existing literature has backed RBF as the best kernel function due its optimization efficiency and adaptability^1,13^. After trial and error, it was indeed determined that RBF produced the best SF predictions, hence it was chosen and finalized as the SVR kernel function in the present study. All other unmentioned SVR hyperparameters were remained as their default values as satisfactory SF predictions were obtained. Table 7 shows the hyperparameter tuning for SVR in the present study.

**Table 7:** Hyperparameter tuning for SVR algorithm.

Hyperparameter tuning of SVR algorithm		
Hyperparameter	Selected	Default
Kernel function	RBF	RBF

#### Artificial neural network (ANN)

The ANN is a deep learning algorithm invented based on the neural connections that occur in the biological functions of the human brain^33^. This algorithm essentially comprises of three layers, which are the input layer, hidden layer, and output layer^26,27,33^. The ANN architecture consists of processing units called neurons, also referred to as nodes^26^. The ANN layers and nodes are connected together by connections referred to as weights^26,27^. These weights provide the ANN with a high degree of flexibility, giving it the ability to freely adapt to input data^27^. The number of ANN layers and nodes required to solve a prediction problem typically depends on the complexity of the problem, with more difficult problems usually requiring more layers or nodes. An ANN architecture is essentially characterized by the work of a training algorithm to represent the layers, nodes, and connections; connection weights between each neuron; and an activation function^26^. The training algorithms works to reduce errors through the adjustment of connection weights and biases within an ANN architecture. The adjusted connection weights are then taken and multiplied with the input values, which are then added with the adjusted biases. Finally, the outputs are sent to the activation function to generate the final output, which in the present study is SF prediction. As explained by Zakaria et al.^26^, the ANN mathematical model can be described by equation:

3 $${y}_{i}=f\left(\sum_{i=1}^{N}{\omega }_{ij}{x}_{i}+{b}_{j}\right)$$

where ${y}_{i}$ is the output variable, *N* is the number of neurons, ${\omega }_{ij}$ is the weight connecting the *jth* neuron and the *ith* neuron, ${x}_{i}$ is the input vector, *b*_*j*_ is the bias of the *jth* neuron, and *f* is the activation function.

As explained by Zamanisabzi et al.^33^, trial-and-error is needed to determine the best hyperparameter tuning for an ANN architecture, as different problems have different hidden relationships within the data. After performing the trial-and-errors, it was determined that two hidden layers with 6 neurons in each layer was optimal for SF prediction in the present study as it provided good adaptability in producing SF predictions for the 11 different river data sets. In addition, different number of epochs, training algorithms, activation functions, and batch numbers were tested to discover the best possible ANN architecture within the context of the present study. Through the testing, the best ANN architecture was found and is shown in Table 8. All other unmentioned ANN hyperparameters including initializer, regularizer, and constraints, were remained as their default values as satisfactory SF predictions were obtained.

**Table 8:** Hyperparameter tuning for ANN algorithm.

Hyperparameter tuning of ANN algorithm		
Hyperparameter	Selected	Default
Number of hidden layers	2	No default
Number of neurons in each hidden layer	6	No default
Number of epochs	100	1
Early callback function	When validation loss does not improve after 50 epochs	None
Batch number	32	32
Training algorithm	Adam	RMSprop
Activation function	ReLU	None
Loss function	MSE	No default

**Figure 4:**
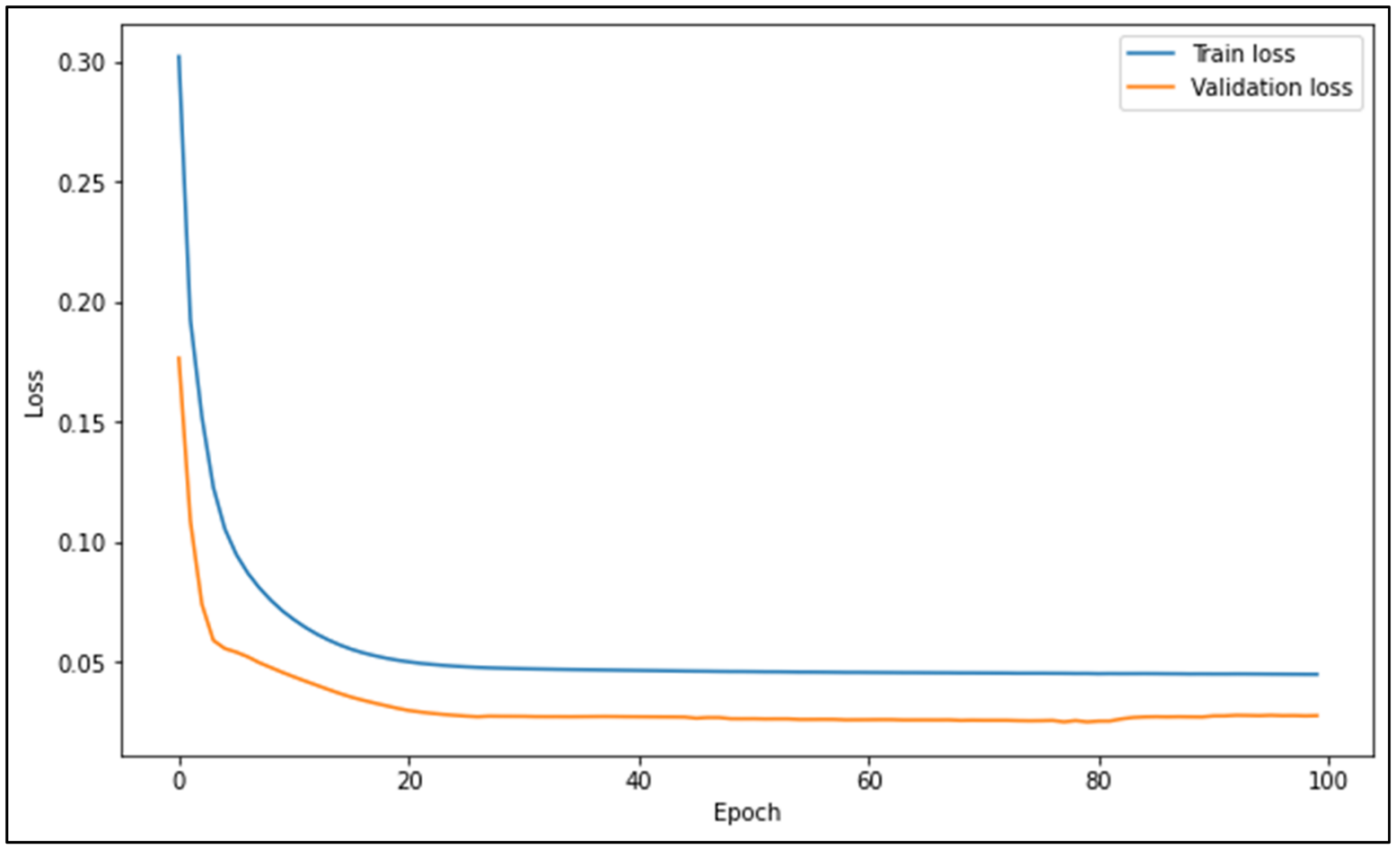
Train and validation loss vs epochs for ANN3 model training process.

During each of the ANN models’ training process, the train and validation loss vs epochs graphs are produced to graphically verify that the losses reduce and converge, and to ensure that overfitting does not occur. As a sample, the losses vs epochs graph for the best performing ANN model (ANN3) for the Johor data set is shown in Fig. 4. It can be seen that the validation loss is lesser than the train loss. This is because of the small size of the validation set, which comprises of 20% of the training set. The size of the validation set can be increased to reduce the train loss; however, it was found that the best SF predictions were obtained with the training data to validation data ratio set at 80:20. Therefore, this ratio was maintained and utilized in training the ANN models.

#### Long short-term memory (LSTM)

The LSTM is an advanced version of the recurrent neural network (RNN) that helps to overcome the issues of gradient vanishing and explosion that are present in the standalone RNN^44^. This algorithm utilizes control gates to essentially store, remove, update, and control the flow of information in a unique structure known as the memory cell^43,44^. There are three types of control gates used by the LSTM, which are the input gate, the output gate, and the control gate^42–44^. The input gate functions to control the flow of information to be introduced into the cell state, the output gate selects information from the cell state to be forwarded to a dense layer containing a single neuron where the final output value is calculated, while the forget gate determines the amount of information to be removed from the previous cell state^43,44^. The operation of the control gates helps in filtering relevant information as required, hence contributing towards the minimization of errors. As mentioned by existing studies^43,44^, the LSTM mathematical model can be described through function:

4 $${h}_{t}={o}_{t}{\odot \mathrm{tanh}(C}_{t})$$

where *h*_*t*_ is the output, *o*_*t*_ is the output gate, $\odot$ is the Hadamard product, and *C*_t_ is the cell status value at time *t*.

As is the case with ANNs, LSTMs also consist of hidden layers filled with neurons, hence a trial-and-error process is needed to find the optimal number of hidden layers and neurons. After performing the trial-and-errors, it was determined that two hidden layers with 50 neurons in each layer was optimal for SF prediction in the present study as it provided good adaptability in producing SF predictions for the 11 different river data sets. In addition, different number of epochs, step numbers, training algorithm, dropout regularization on each hidden layer, activation function, recurrent activation function, and batch numbers, were tested to discover the best possible LSTM architecture within the context of the present study. Through the testing, the best LSTM architecture was found and is shown in Table 9. All other unmentioned LSTM hyperparameters including initializer, regularizer, and constraints, were remained as their default values as satisfactory SF predictions were obtained.

**Table 9:** Hyperparameter tuning of LSTM algorithm.

Hyperparameter tuning of LSTM algorithm		
Hyperparameter	Selected	Default
Number of hidden layers	2	No default
Number of neurons in each hidden layer	50	No default
Number of epochs	100	1
Early callback function	When validation loss does not improve after 50 epochs	None
Step number	7	No default
Batch number	32	32
Training algorithm	Adam	RMSprop
Dropout regularization on each hidden layer	0.2	None
Activation function	tanh	tanh
Recurrent activation function	sigmoid	sigmoid
Loss function	MSE	No default

**Figure 5:**
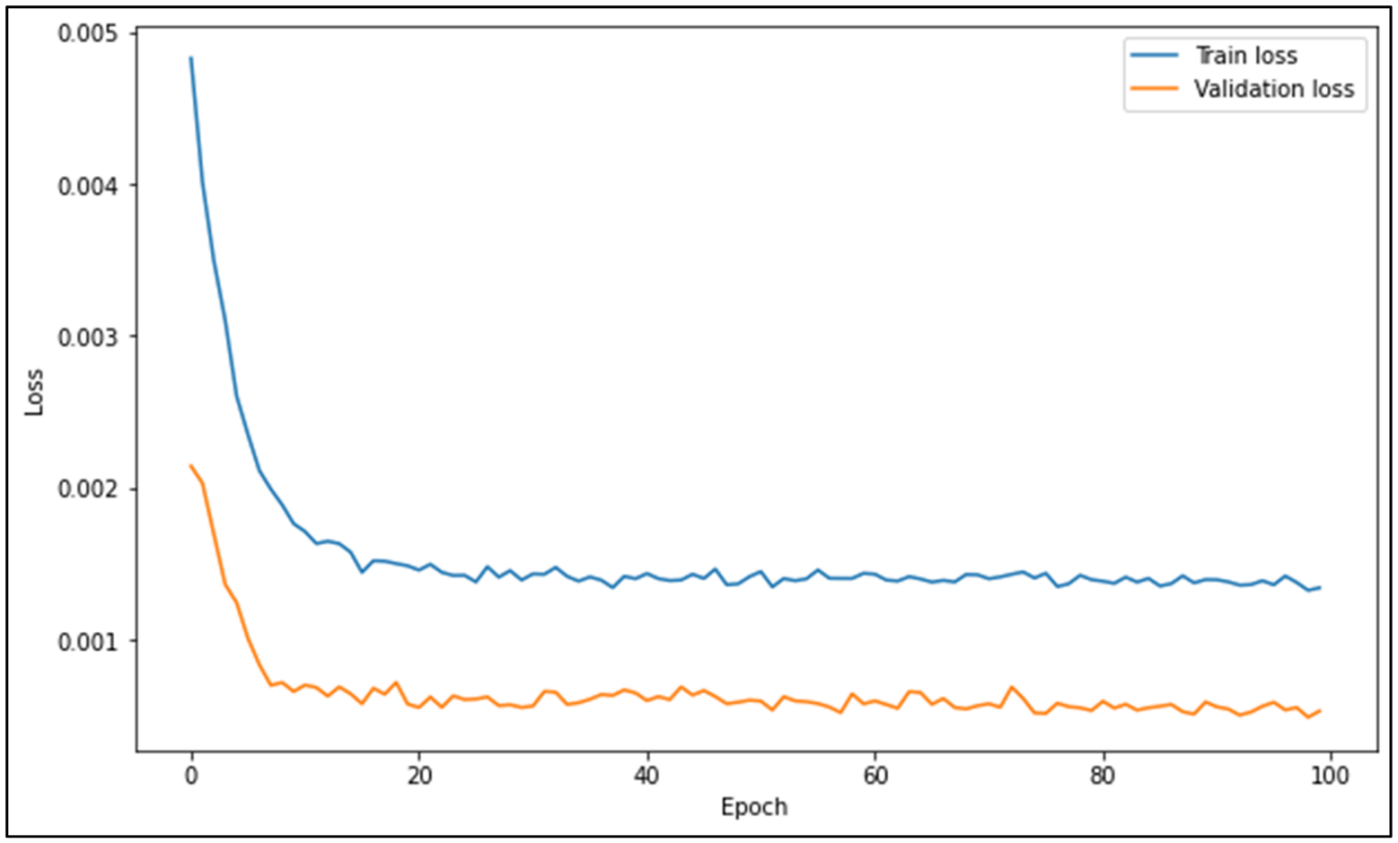
Train and validation loss vs epochs for LSTM2 model training process.

During each of the LSTM models’ training process, the train and validation loss vs epochs graphs are produced to graphically verify that the losses reduce and converge, and to ensure that overfitting does not occur. As a sample, the losses vs epochs graph for the best performing LSTM model (LSTM2) for the Johor data set is shown in Fig. 5. It can be seen that the validation loss is lesser than the train loss, similar to Fig. 4. This is because of the small size of the validation set, which comprises of 20% of the training set. The size of the validation set can be increased to reduce the train loss; however, it was found that the best SF predictions were obtained with the training data to validation data ratio set at 80:20. Therefore, this ratio was maintained and utilized in training the ANN models. Additionally, the higher train loss may be due to the dropout regularization applied in the LSTM model structure. The dropout regularization was applied to reduce validation loss, hence leading to better generalization outside the validation and test sets. However, the dropout regularization may sacrifice train accuracy to enhance validation accuracy, which may cause train loss to be higher than validation loss. On top of that, regularization methods are only applied during training and not during validation, which can also cause train loss to be higher than validation loss.

### Performance measures

Four performance measures were utilized to evaluate the SF prediction models’ performances, namely the mean absolute error (MAE), root mean squared error (RMSE), coefficient of determination (R^2^), and ranking mean (RM). MAE, RMSE, and R^2^ have been frequently used in existing SF prediction studies^4,13–15,18,34,43,54,59^. RM was utilized by Ahmed et al.^60^ as a means to rank overall model performance.

#### Mean absolute error (MAE)

The MAE calculates the average absolute difference between predicted and actual values; hence a lower MAE is desired. The MAE is measured cubic meters per second (m^3^/s) in the present study. MAE is calculated by:

5 $$MAE=\frac{1}{n}\cdot \left[\sum_{i=1}^{n}\left|{y}_{i}-\widehat{{y}_{i}}\right|\right]$$

where ${y}_{i}$ is the real value, $\widehat{{y}_{i}}$ is the predicted value, and $n$ is the sample size.

#### Root mean squared error (RMSE)

The RMSE is a metric that places a relatively high weight on large errors, hence making it a useful indicator of large errors. A lower RMSE is typically desired. In the present study, the RMSE is measured in units of cubic meters per second (m^3^/s). The following equation is used for the computation of RMSE:

6 $$RMSE=\sqrt{\frac{1}{n}\cdot \left[\sum_{i=1}^{n}{\left({y}_{i}-\widehat{{y}_{i}}\right)}^{2}\right]}$$

where ${y}_{i}$ is the real value, $\widehat{{y}_{i}}$ is the predicted value, and $n$ is the sample size.

#### Coefficient of determination (R^2^)

The R^2^ computes the correlation between real values and predicted values, with the range of R^2^ scores between − 1 and 1. An R^2^ closer to 1 signals a high correlation between real and predicted values. R^2^ scores are unitless. The following equation is used to calculate R^2^:

7 $${R}^{2}=1-\left[\frac{\sum_{i=1}^{n}{\left({y}_{i}-\widehat{{y}_{i}}\right)}^{2}}{\sum_{i=1}^{n}{\left({y}_{i}-\overline{{y }_{i}}\right)}^{2}}\right]$$

where ${y}_{i}$ is real value, $\widehat{{y}_{i}}$ is predicted value, $\overline{{y }_{i}}$ is the mean of ${y}_{i}$, and $n$ is sample size.

#### Ranking mean (RM)

To compute the RM, each model is first ranked based on the scores of the selected performance measures, which are MAE, RMSE, and R^2^ in the present study. Each models’ RM is then calculated by obtaining the average of their ranks respective to their MAE, RMSE and R^2^ scores. A higher RM signals a better overall performance of a model compared to the other models. RM is defined by:

8 $$RM=\frac{1}{n}\sum_{i=1}^{n}{rank}_{i}$$

where $n$ is the number of performance evaluation measures used, which is 3.

## Results and discussion

This section presents and discusses the performances of the developed models for SSL prediction. A comparison and analysis is then made based on the model performances.

### Performance of models based on the Sungai Johor, Johor data set

**Figure 6:**
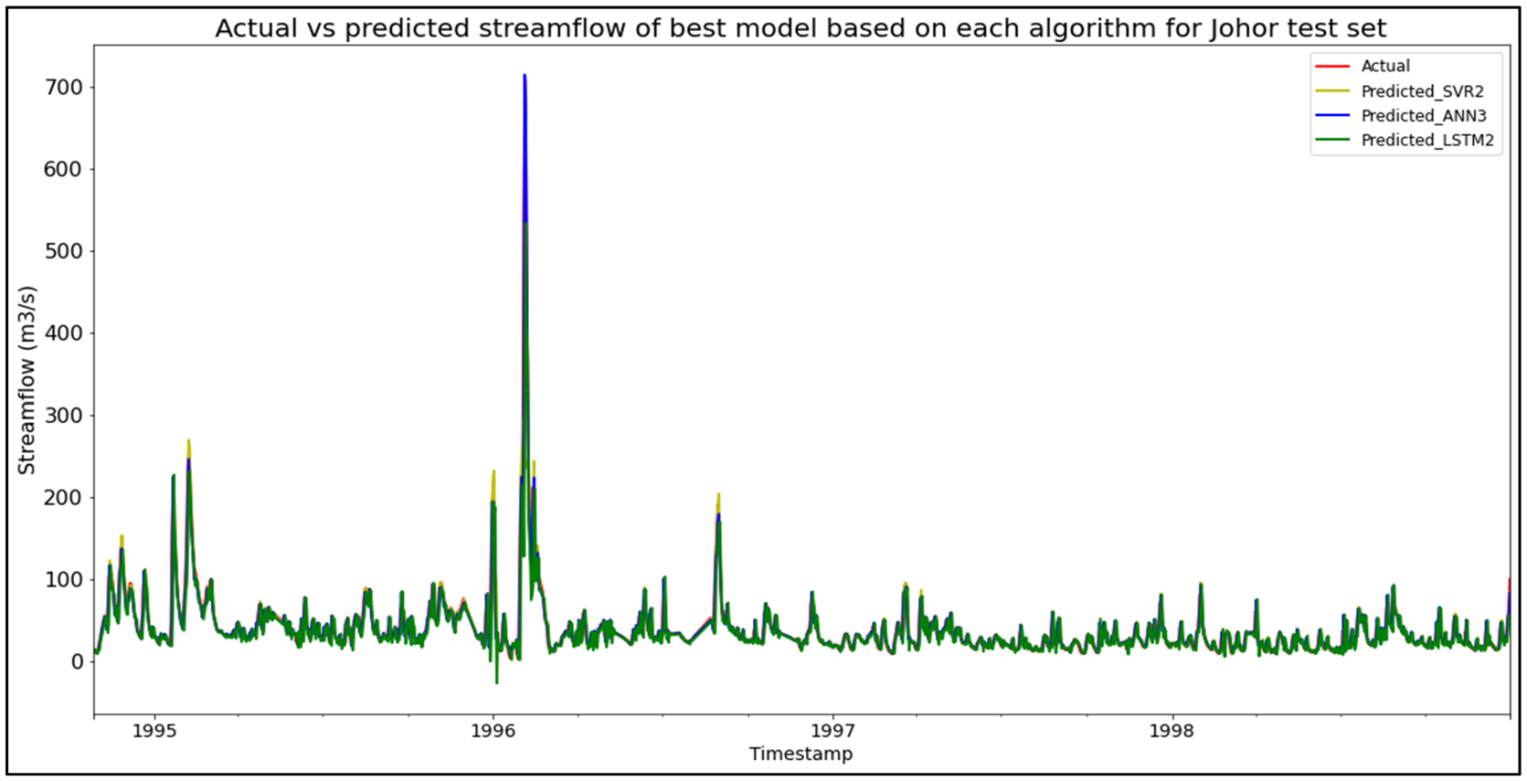
Actual vs predicted SF of best models based on each algorithm for Sungai Johor test set.

The best overall performance in predicting SF for the Sungai Johor, Johor data set was produced by model ANN3, which is based on the ANN algorithm and input parameter scenario 3. ANN3 outperformed the other models with MAE, RMSE, and R^2^ scores of 4.7235 m^3^/s, 10.0746 m^3^/s, and 0.9443 respectively, hence obtaining the highest RM with a score of 1.00. SVR2 was the best SVR model (RM = 4.00), while LSTM2 was the best LSTM model (RM = 7.00). The models’ performance scores and actual vs predicted SF of best models based on each algorithm for the Sungai Johor test set are shown in Table 10 and Fig. 6 respectively.

**Table 10:** Models’ performance scores based on Sungai Johor test set.

Model	MAE	RMSE	R^2^	Rank (MAE)	Rank (RMSE)	Rank (R^2^)	RM
SVR1	6.6855	21.3127	0.7509	6	6	6	6.00
SVR2	5.6855	20.7216	0.7645	4	4	4	4.00
SVR3	5.5750	21.1976	0.7536	3	5	5	4.33
ANN1	6.0686	14.3558	0.8870	5	3	3	3.67
ANN2	4.8265	10.9228	0.9346	2	2	2	2.00
**ANN3**	**4.7235**	**10.0746**	**0.9443**	**1**	**1**	**1**	**1.00**
LSTM1	9.3446	21.7108	0.7421	8	8	8	8.00
LSTM2	9.3061	21.6780	0.7429	7	7	7	7.00
LSTM3	9.5766	22.1443	0.7317	9	9	9	9.00

Significant values are in bold.

### Performance of models based on the Sungai Muda, Kedah data set

**Figure 7:**
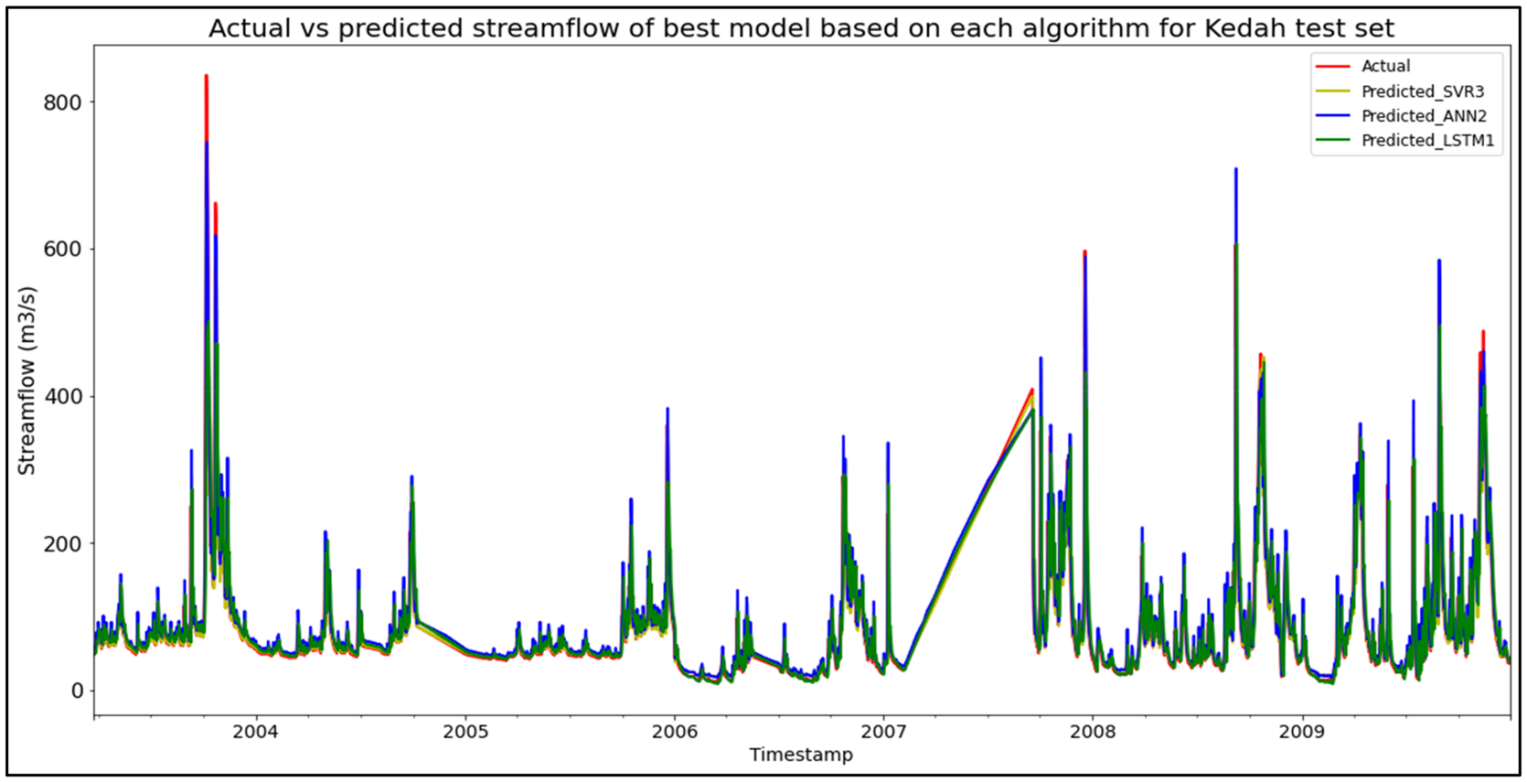
Actual vs predicted SF of best models based on each algorithm for Sungai Muda test set.

Model SVR3, based on the SVR algorithm and input parameter scenario 3, produced the best overall performance in predicting SF for the Sungai Muda, Kedah data set. SVR3 significantly outperformed the other models in terms of MAE with a score of 12.3853 m^3^/s, hence obtaining the best RM with a score of 1.67. ANN2 achieved the best RMSE and R^2^ with scores of 29.6536 m^3^/s and 0.8911 respectively. ANN2 was the best ANN model (RM = 2.67), while LSTM1 was the best LSTM model (RM = 7.00). The models’ performance scores and actual vs predicted SF of best models from each algorithm for the Sungai Muda test set are shown in Table 11 and Fig. 7 respectively.

**Table 11:** Models’ performance scores based on Sungai Muda test set.

Model	MAE	RMSE	R^2^	Rank (MAE)	Rank (RMSE)	Rank (R^2^)	RM
SVR1	13.3065	30.8601	0.8821	3	6	6	5.00
SVR2	13.0296	30.2442	0.8867	2	4	4	3.33
**SVR3**	**12.3853**	29.7585	0.8903	**1**	2	2	**1.67**
ANN1	14.6595	30.3473	0.8859	5	5	5	5.00
ANN2	15.4957	**29.6536**	**0.8911**	6	**1**	**1**	2.67
ANN3	14.4053	29.7719	0.8902	4	3	3	3.33
LSTM1	20.4944	45.3718	0.7456	7	7	7	7.00
LSTM2	22.3298	45.7841	0.7409	8	8	8	8.00
LSTM3	22.8569	45.9370	0.7392	9	9	9	9.00

Significant values are in bold.

### Performance of models based on the Sungai Kelantan, Kelantan data set

**Figure 8:**
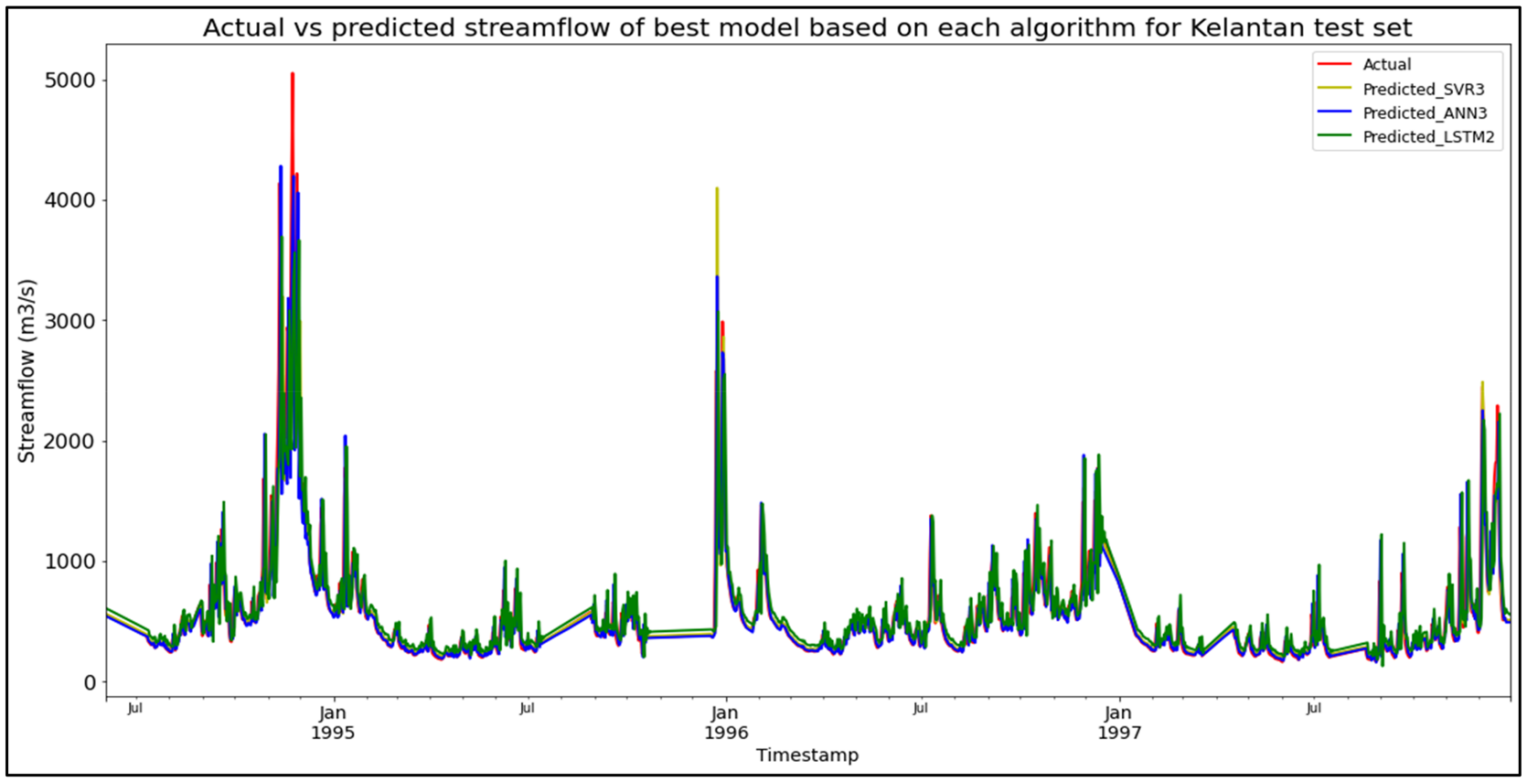
Actual vs predicted SF of best models based on each algorithm for Sungai Kelantan test set.

The best overall performance in predicting SF for the Sungai Kelantan, Kelantan data set was produced by model SVR3, which is based on the SVR algorithm and input parameter scenario 3. SVR3 outperformed the other models with MAE, RMSE, and R^2^ scores of 73.0989 m^3^/s, 173.7072 m^3^/s, and 0.8529 respectively, hence obtaining the highest RM with a score of 1.00. ANN3 was the best ANN model (RM = 2.67), while LSTM2 was the best LSTM model (RM = 7.33). The models’ performance scores and actual vs predicted SF of best models based on each algorithm for the Sungai Kelantan test set are shown in Table 12 and Fig. 8 respectively.

**Table 12:** Models’ performance scores based on Sungai Kelantan test set.

Model	MAE	RMSE	R^2^	Rank (MAE)	Rank (RMSE)	Rank (R^2^)	RM
SVR1	75.2411	175.7898	0.8493	4	4	4	4.00
SVR2	73.9793	176.0282	0.8489	3	5	5	4.33
**SVR3**	**73.0989**	**173.7072**	**0.8529**	**1**	**1**	**1**	**1.00**
ANN1	76.3966	174.9986	0.8507	6	2	2	3.33
ANN2	75.8246	180.5615	0.8410	5	6	6	5.67
ANN3	73.7096	175.1687	0.8504	2	3	3	2.67
LSTM1	133.7528	265.5139	0.6580	7	9	9	8.33
LSTM2	134.1845	264.5478	0.6605	8	7	7	7.33
LSTM3	134.3691	264.5587	0.6605	9	8	8	8.33

Significant values are in bold.

### Performance of models based on the Sungai Melaka, Melaka data set

**Figure 9:**
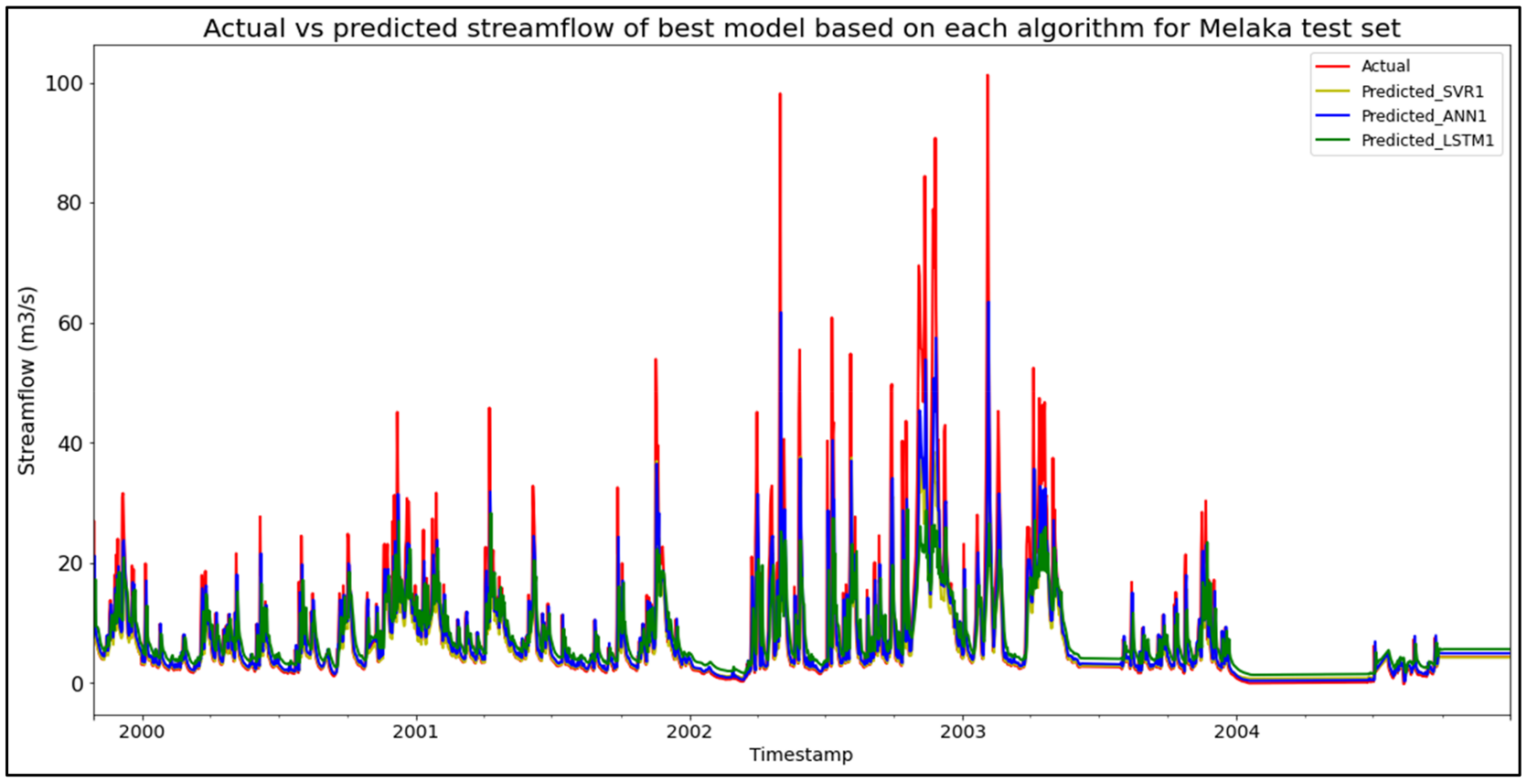
Actual vs predicted SF of best models based on each algorithm for Sungai Melaka test set.

The best overall performance in predicting SF for the Sungai Melaka, Melaka data set was produced by model ANN1, which is based on the ANN algorithm and input parameter scenario 1. ANN1 outperformed the other models with MAE, RMSE, and R^2^ scores of 2.7113 m^3^/s, 6.0824 m^3^/s, and 0.6809 respectively, hence obtaining the highest RM with a score of 1.00. SVR1 was the best SVR model (RM = 3.67), while LSTM1 was the best LSTM model (RM = 7.67). The models’ performance scores and actual vs predicted SF of best models based on each algorithm for the Sungai Melaka test set are shown in Table 13 and Fig. 9 respectively.

**Table 13:** Models’ performance scores based on Sungai Melaka test set.

Model	MAE	RMSE	R^2^	Rank (MAE)	Rank (RMSE)	Rank (R^2^)	RM
SVR1	2.7688	6.7611	0.6057	3	4	4	3.67
SVR2	2.9154	7.3515	0.5339	5	5	5	5.00
SVR3	2.9217	7.5694	0.5058	6	6	6	6.00
**ANN1**	**2.7113**	**6.0824**	**0.6809**	**1**	**1**	**1**	**1.00**
ANN2	2.8758	6.5140	0.6340	4	3	3	3.33
ANN3	2.7218	6.1571	0.6730	2	2	2	2.00
LSTM1	4.1403	8.1246	0.4327	9	7	7	7.67
LSTM2	4.0984	8.1950	0.4228	7	9	9	8.33
LSTM3	4.1347	8.1269	0.4324	8	8	8	8.00

Significant values are in bold.

### Performance of models based on the Sungai Kepis, Negeri Sembilan data set

**Figure 10:**
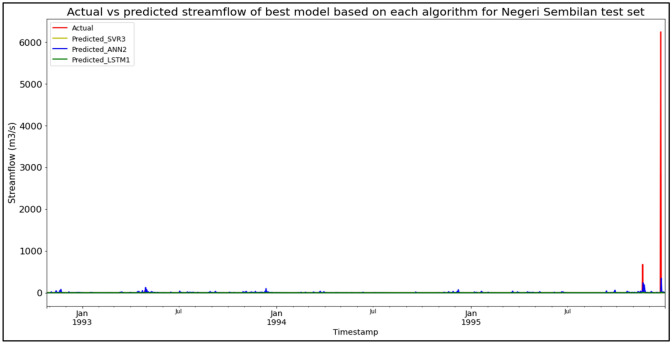
Actual vs predicted SF of best models based on each algorithm for Sungai Kepis test set.

The best overall performance in predicting SF for the Sungai Kepis, Negeri Sembilan data set was produced by model LSTM3, which is based on the LSTM algorithm and input parameter scenario 3. LSTM3 outperformed the other models with MAE, RMSE, and R^2^ scores of 0.4969 m^3^/s, 2.6430 m^3^/s, and 0.0202 respectively, hence obtaining the highest RM with a score of 1.00. SVR1 and SVR2 were the joint-best SVR models (RM = 4.67), while ANN2 was the best ANN model (RM = 7.00). The models’ performance scores and actual vs predicted SF of best models based on each algorithm for the Sungai Kepis test set are shown in Table 14 and Fig. 10 respectively.

**Table 14:** Models’ performance scores based on Sungai Kepis test set.

Model	MAE	RMSE	R^2^	Rank (MAE)	Rank (RMSE)	Rank (R^2^)	RM
SVR1	8.8078	183.6942	0.0001	5	6	6	5.67
SVR2	8.8279	183.6641	0.0004	6	4	4	4.67
SVR3	8.7211	183.6811	0.0002	4	5	5	4.67
ANN1	10.5674	183.8572	− 0.0017	8	8	8	8.00
ANN2	10.5064	183.7476	− 0.0005	7	7	7	7.00
ANN3	10.8344	183.9346	− 0.0025	9	9	9	9.00
LSTM1	0.5061	2.6564	0.0102	3	2	2	2.33
LSTM2	0.5028	2.6654	0.0035	2	3	3	2.67
**LSTM3**	**0.4969**	**2.6430**	**0.0202**	**1**	**1**	**1**	**1.00**

Significant values are in bold.

### Performance of models based on the Sungai Pahang, Pahang data set

**Figure 11:**
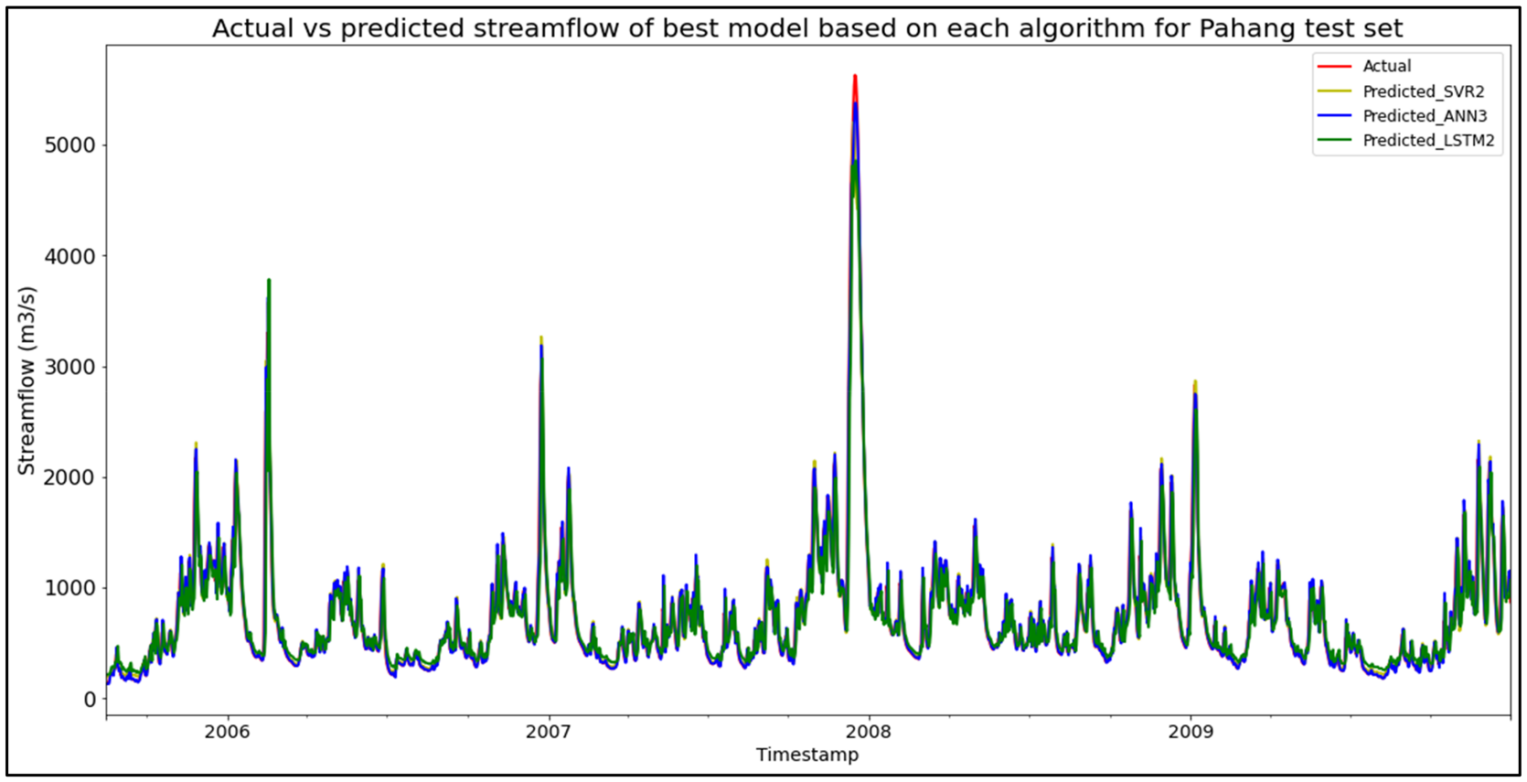
Actual vs predicted SF of best models based on each algorithm for Sungai Pahang test set.

The best overall performance in predicting SF for the Sungai Pahang, Pahang data set was produced by model ANN3, which is based on the ANN algorithm and input parameter scenario 3. ANN3 outperformed the other models with MAE, RMSE, and R^2^ scores of 59.0621 m^3^/s, 100.9960 m^3^/s, and 0.9700 respectively, hence obtaining the highest RM with a score of 1.00. SVR2 was the best SVR model (RM = 3.33), while LSTM2 was the best LSTM model (RM = 7.33). The models’ performance scores and actual vs predicted SF of best models based on each algorithm for the Sungai Pahang test set are shown in Table 15 and Fig. 11 respectively.

**Table 15:** Models’ performance scores based on Sungai Pahang test set.

Model	MAE	RMSE	R^2^	Rank (MAE)	Rank (RMSE)	Rank (R^2^)	RM
SVR1	85.0020	143.9947	0.9389	6	6	6	6.00
SVR2	68.8680	118.2528	0.9588	4	3	3	3.33
SVR3	65.5277	120.8079	0.9570	3	4	4	3.67
ANN1	81.6888	137.3886	0.9444	5	5	5	5.00
ANN2	62.7678	105.4579	0.9672	2	2	2	2.00
**ANN3**	**59.0621**	**100.9960**	**0.9700**	**1**	**1**	**1**	**1.00**
LSTM1	130.0995	215.9988	0.8625	9	9	9	9.00
LSTM2	128.5396	213.9796	0.8651	8	7	7	7.33
LSTM3	127.6468	214.9783	0.8638	7	8	8	7.67

Significant values are in bold.

### Performance of models based on the Sungai Perak, Perak data set

**Figure 12:**
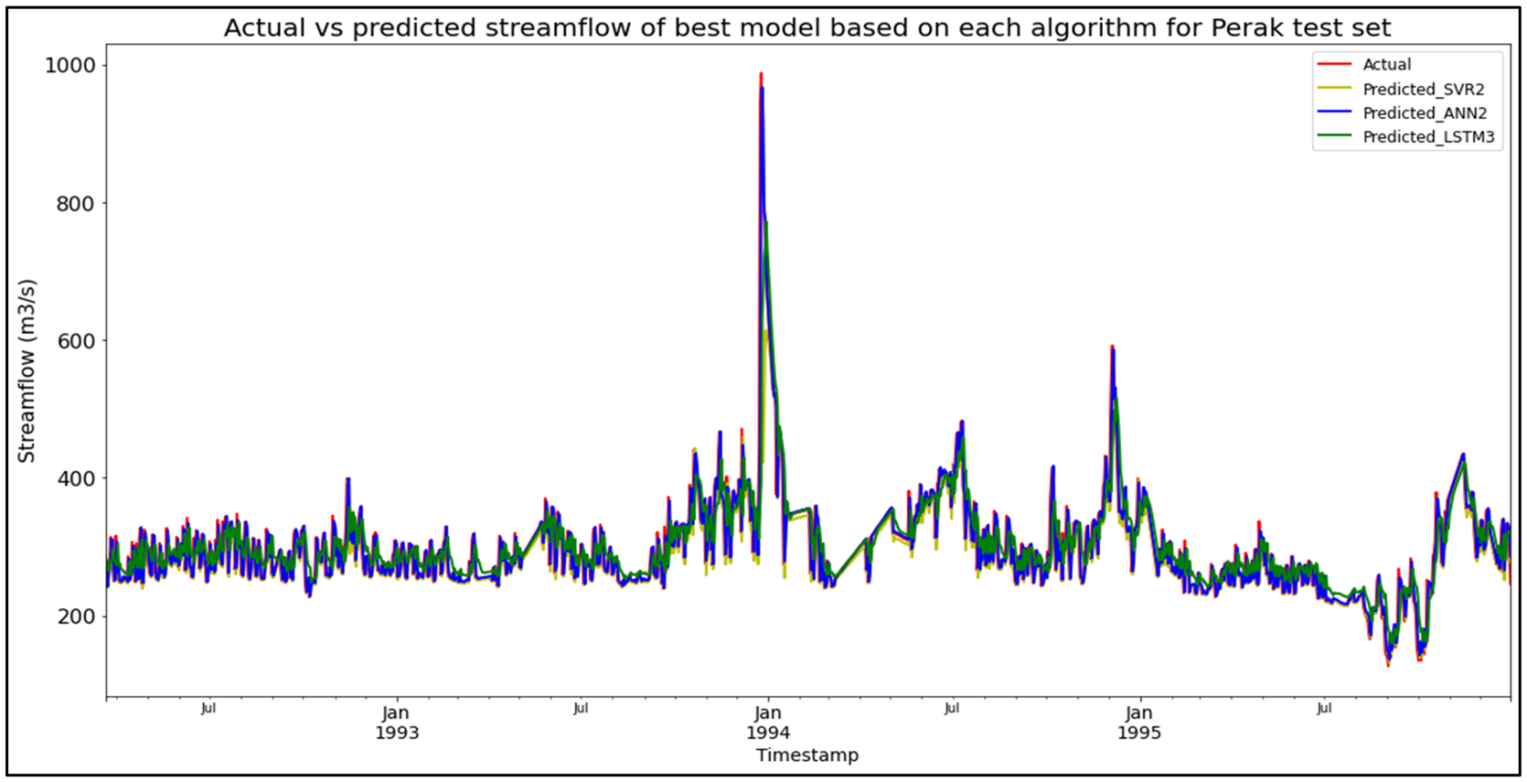
Actual vs predicted SF of best models based on each algorithm for Sungai Perak test set.

The best overall performance in predicting SF for the Sungai Perak, Perak data set was produced by model ANN2, which is based on the ANN algorithm and input parameter scenario 2. ANN2 outperformed the other models with MAE, RMSE, and R^2^ scores of 18.1337 m^3^/s, 29.3009 m^3^/s, and 0.8286 respectively, hence obtaining the highest RM with a score of 1.00. SVR2 was the best SVR model (RM = 4.33), while LSTM3 was the best LSTM model (RM = 7.00). The models’ performance scores and actual vs predicted SF of best models based on each algorithm for the Sungai Perak test set are shown in Table 16 and Fig. 12 respectively.

**Table 16:** Models’ performance scores based on Sungai Perak test set.

Model	MAE	RMSE	R^2^	Rank (MAE)	Rank (RMSE)	Rank (R^2^)	RM
SVR1	19.8748	36.5196	0.7338	6	4	4	4.67
SVR2	19.6637	37.3820	0.7211	3	5	5	4.33
SVR3	19.7318	39.0645	0.6954	4	6	6	5.33
ANN1	19.7860	30.5695	0.8135	5	3	3	3.67
**ANN2**	**18.1337**	**29.3009**	**0.8286**	**1**	**1**	**1**	**1.00**
ANN3	18.2248	29.7523	0.8233	2	2	2	2.00
LSTM1	25.4832	40.5151	0.6736	9	8	8	8.33
LSTM2	25.4778	40.6169	0.6719	8	9	9	8.67
LSTM3	25.0893	40.4116	0.6752	7	7	7	7.00

Significant values are in bold.

### Performance of models based on the Sungai Arau, Perlis data set

**Figure 13:**
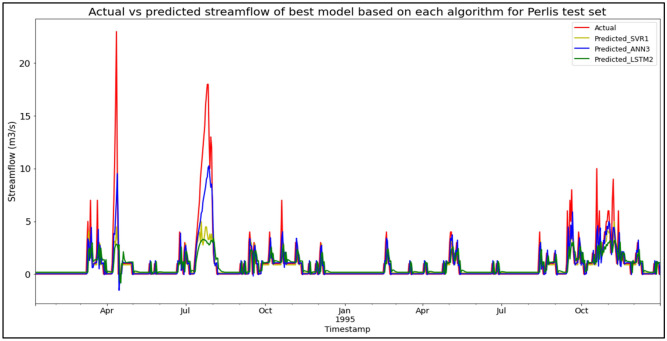
Actual vs predicted SF of best models based on each algorithm for Sungai Arau test set.

The best overall performance in predicting SF for the Sungai Arau, Perlis data set was produced by model ANN3, which is based on the ANN algorithm and input parameter scenario 3. ANN3 outperformed the other models with MAE, RMSE, and R^2^ scores of 0.5441 m^3^/s, 1.4007 m^3^/s, and 0.6857 respectively, hence obtaining the highest RM with a score of 1.00. SVR1 was the best SVR model (RM = 4.00), while LSTM2 was the best LSTM model (RM = 7.00). The models’ performance scores and actual vs predicted SF of best models based on each algorithm for the Sungai Arau test set are shown in Table 17 and Fig. 13 respectively.

**Table 17:** Models’ performance scores based on Sungai Arau test set.

Model	MAE	RMSE	R^2^	Rank (MAE)	Rank (RMSE)	Rank (R^2^)	RM
SVR1	0.6810	1.8868	0.4296	4	4	4	4.00
SVR2	0.7022	2.0166	0.3485	5	5	5	5.00
SVR3	0.7143	2.0696	0.3138	6	6	6	6.00
ANN1	0.5626	1.5062	0.6365	2	2	2	2.00
ANN2	0.5674	1.5568	0.6117	3	3	3	3.00
**ANN3**	**0.5441**	**1.4007**	**0.6857**	**1**	**1**	**1**	**1.00**
LSTM1	0.8803	2.2061	0.2264	9	9	9	9.00
LSTM2	0.8595	2.0975	0.3007	7	7	7	7.00
LSTM3	0.8610	2.1073	0.2941	8	8	8	8.00

Significant values are in bold.

### Performance of models based on the Sungai Selangor, Selangor data set

**Figure 14:**
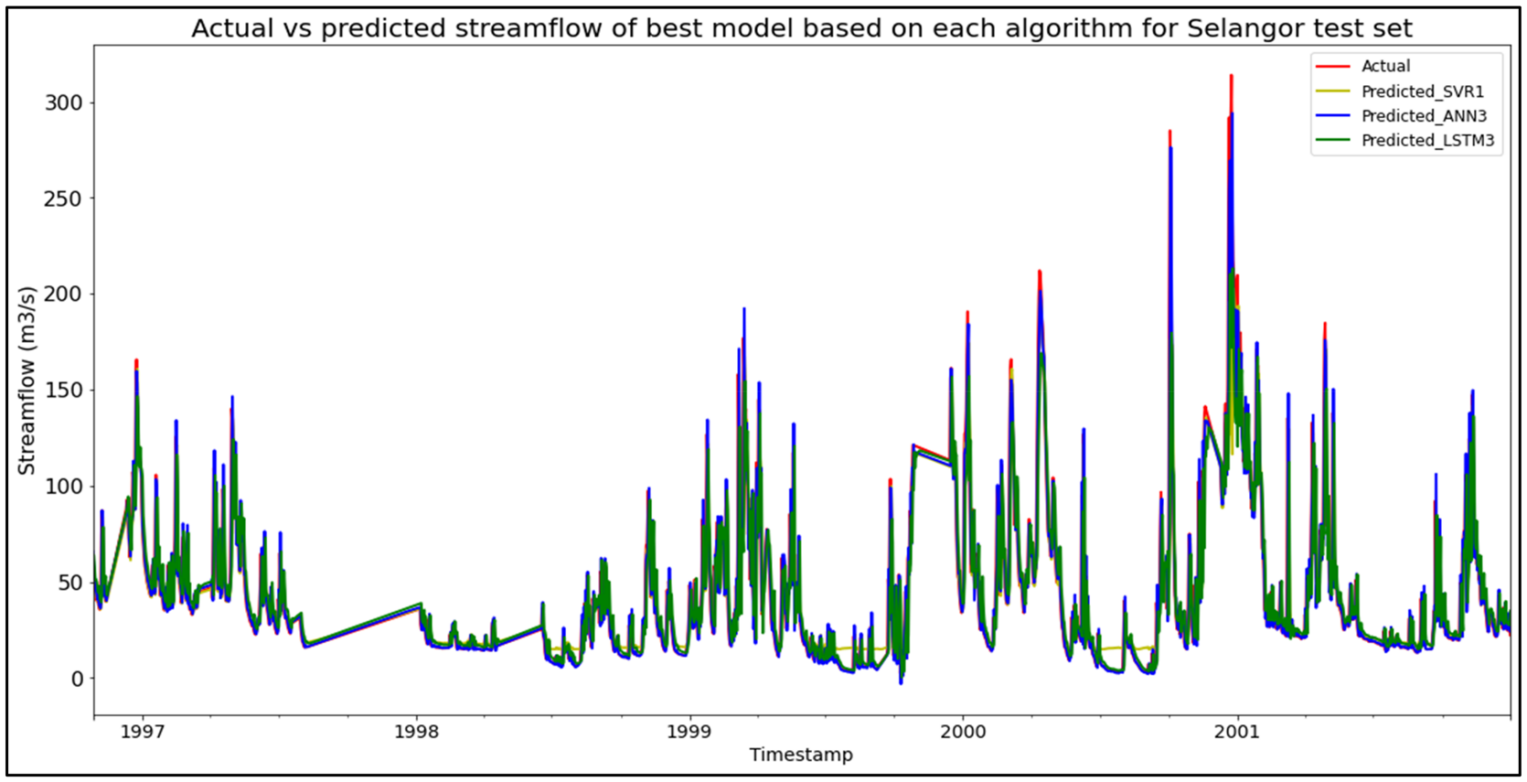
Actual vs predicted SF of best models based on each algorithm for Sungai Selangor test set.

The best overall performance in predicting SF for the Sungai Selangor, Selangor data set was produced by model ANN3, which is based on the ANN algorithm and input parameter scenario 3. ANN3 outperformed the other models with MAE, RMSE, and R^2^ scores of 7.2175 m^3^/s, 13.9196 m^3^/s, and 0.8851 respectively, hence obtaining the highest RM with a score of 1.00. SVR1 was the best SVR model (RM = 4.67), while LSTM3 was the best LSTM model (RM = 7.00). The models’ performance scores and actual vs predicted SF of best models based on each algorithm for the Sungai Selangor test set are shown in Table 18 and Fig. 14 respectively.

**Table 18:** Models’ performance scores based on Sungai Selangor test set.

Model	MAE	RMSE	R^2^	Rank (MAE)	Rank (RMSE)	Rank (R^2^)	RM
SVR1	8.6747	15.7719	0.8525	6	4	4	4.67
SVR2	8.6363	16.2904	0.8426	5	5	5	5.00
SVR3	8.4787	16.4230	0.8400	4	6	6	5.33
ANN1	7.8569	14.1281	0.8816	3	3	3	3.00
ANN2	7.3758	13.9850	0.8840	2	2	2	2.00
**ANN3**	**7.2175**	**13.9196**	**0.8851**	**1**	**1**	**1**	**1.00**
LSTM1	11.2372	20.2553	0.7551	8	9	9	8.67
LSTM2	11.2378	20.2182	0.7560	9	8	8	8.33
LSTM3	11.1610	20.1812	0.7569	7	7	7	7.00

Significant values are in bold.

### Performance of models based on the Sungai Dungun, Terengganu data set

**Figure 15:**
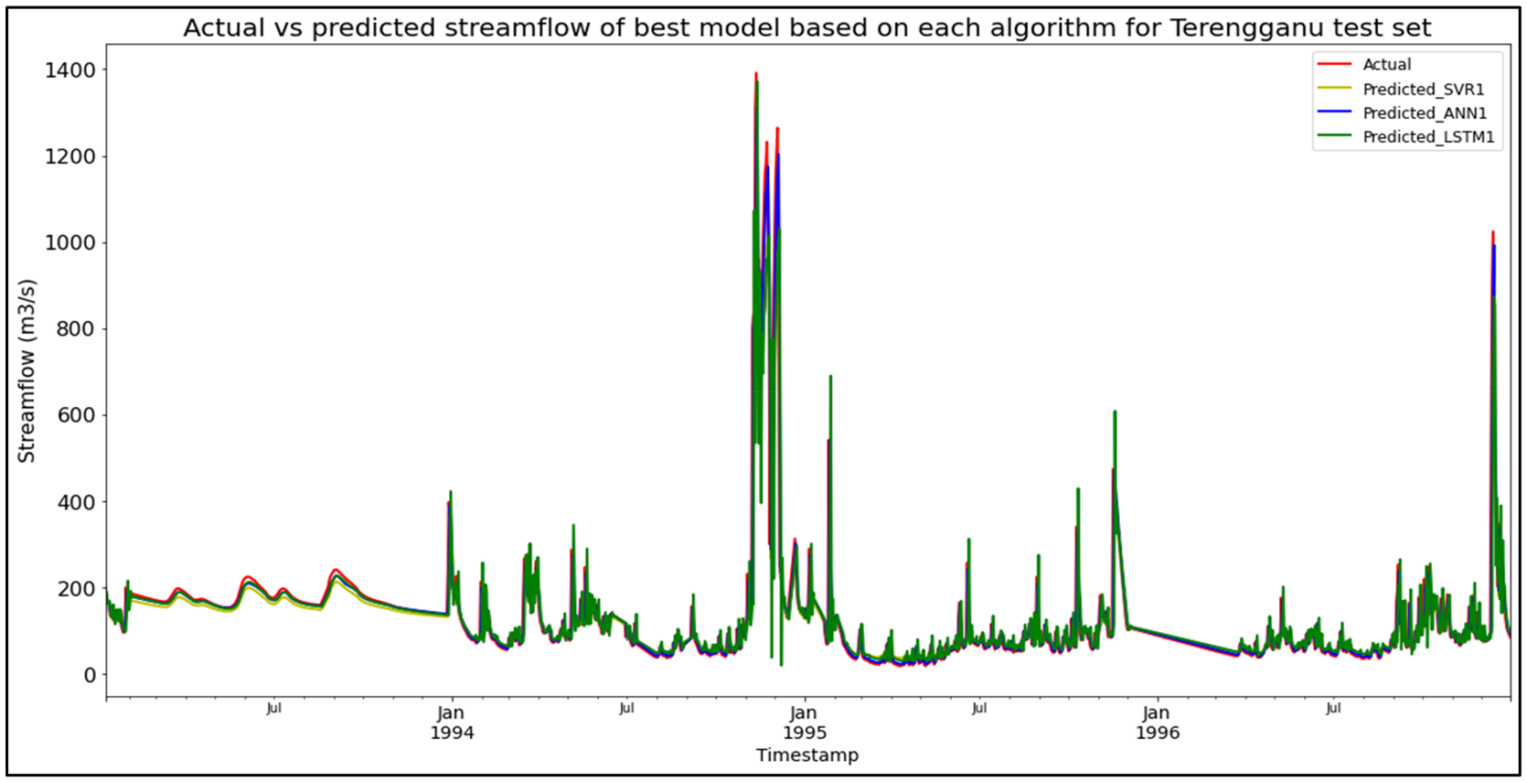
Actual vs predicted SF of best models based on each algorithm for Sungai Dungun test set.

The best overall performance in predicting SF for the Sungai Dungun, Terengganu data set was produced by model ANN1, which is based on the ANN algorithm and input parameter scenario 1. ANN1 outperformed the other models with MAE, RMSE, and R^2^ scores of 18.8022 m^3^/s, 51.8025 m^3^/s, and 0.8631 respectively, hence obtaining the highest RM with a score of 1.00. SVR1 was the best SVR model (RM = 4.00), while LSTM1 was the best LSTM model (RM = 7.00). The models’ performance scores and actual vs predicted SF of best models based on each algorithm for the Sungai Dungun test set are shown in Table 19 and Fig. 15 respectively.

**Table 19:** Models’ performance scores based on Sungai Dungun test set.

Model	MAE	RMSE	R^2^	Rank (MAE)	Rank (RMSE)	Rank (R^2^)	RM
SVR1	22.0572	53.9440	0.8516	6	3	3	4.00
SVR2	21.1469	54.2140	0.8501	5	4	4	4.33
SVR3	20.7256	54.6478	0.8477	4	5	5	4.67
**ANN1**	**18.8022**	**51.8025**	**0.8631**	**1**	**1**	**1**	**1.00**
ANN2	18.9638	52.5147	0.8593	2	2	2	2.00
ANN3	20.3061	55.4433	0.8432	3	6	6	5.00
LSTM1	29.1170	76.3889	0.7024	7	7	7	7.00
LSTM2	29.8838	76.7408	0.6997	8	9	9	8.67
LSTM3	30.0394	76.4696	0.7018	9	8	8	8.33

Significant values are in bold.

### Performance of models based on the Sungai Klang, Kuala Lumpur data set

**Figure 16:**
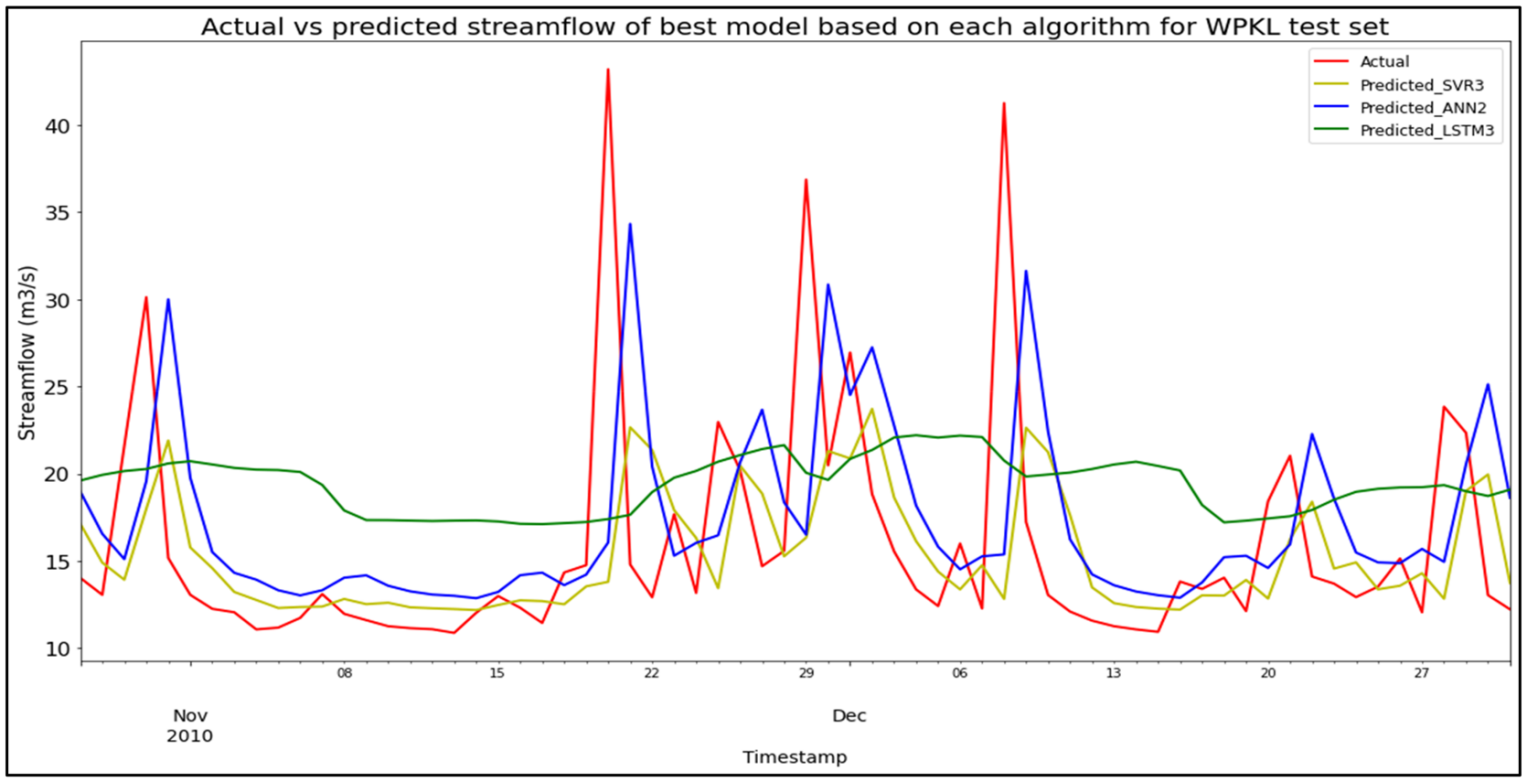
Actual vs predicted SF of best models based on each algorithm for Sungai Klang test set.

Model SVR3, based on the SVR algorithm and input parameter scenario 3, produced the best overall performance in predicting SF for the Sungai Klang, Kuala Lumpur data set. SVR3 outperformed the other models in terms of RMSE and R^2^ with scores of 6.6737 m^3^/s and − 0.0570 respectively, hence obtaining the best RM with a score of 1.33. SVR1 achieved the best MAE with a score of 3.8143 m^3^/s. ANN2 was the best ANN model (RM = 4.67), while LSTM3 was the best LSTM model (RM = 5.67). The models’ performance scores and actual vs predicted SF of best models based on each algorithm for the Sungai Klang test set are shown in Table 20 and Fig. 16 respectively.

**Table 20:** Models’ performance scores based on Sungai Klang test set.

Model	MAE	RMSE	R^2^	Rank (MAE)	Rank (RMSE)	Rank (R^2^)	RM
SVR1	**3.8143**	6.7184	− 0.0712	**1**	2	2	1.67
SVR2	3.9975	6.8580	− 0.1162	3	3	3	3.00
**SVR3**	3.8568	**6.6737**	**− 0.0570**	2	**1**	**1**	**1.33**
ANN1	5.2573	7.5966	− 0.3696	5	5	8	6.00
ANN2	5.0754	7.4943	− 0.3329	4	4	6	4.67
ANN3	6.0143	8.1951	− 0.5939	6	9	9	8.00
LSTM1	6.7049	7.8051	− 0.3499	9	8	7	8.00
LSTM2	6.4877	7.6416	− 0.2939	8	7	5	6.67
LSTM3	6.4529	7.6092	− 0.2830	7	6	4	5.67

Significant values are in bold.

### Overall comparison and discussion of model performances

Two evaluations are considered in comparing and analysing the models’ performances. The first evaluation is the number of times a model produced the best predictive performance for a data set, and the second evaluation is the reliability of each model in producing SF predictions of relatively high accuracy. In the present study, ANN3 produced the best predictive performance for 4 out of the 11 tested data sets (Sungai Johor, Sungai, Sungai Pahang, Sungai Arau, Sungai Selangor). Meanwhile, SVR3 was the most accurate model in 3 out of the 11 tested data sets (Sungai Muda, Sungai Kelantan, Sungai Klang); and ANN1 was the most accurate model in 2 out of the 11 tested data sets (Sungai Melaka, Sungai Dungun). Lastly, ANN2 and LSTM3 achieved the best SF predictions for one data set each, namely Sungai Perak and Sungai Kepis respectively. Overall, it is understood that ANN3 produced the most accurate SF predictive performances for more data sets in comparison to the other tested models. Additional analysis reveals that the algorithm and input scenario that produced the best SF predictive performance for the most data sets are the ANN and input scenario 3 respectively, as they produced the best SF predictions for 7 out of 11 data sets and 8 out of 11 data sets respectively. A matrix of most accurate algorithm and input scenario for each data set and the parameters with highest number of best prediction results can be observed in Tables 21 and 22 respectively.

**Table 21:** Matrix of most accurate algorithm and input scenario for each data set.

Algorithm	Input scenario 1	Input scenario 2	Input scenario 3
SVR			Sungai Muda, Sungai Kelantan, Sungai Klang
ANN	Sungai Melaka, Sungai Dungun	Sungai Perak	Sungai Johor, Sungai Pahang, Sungai Arau, Sungai Selangor
LSTM			Sungai Kepis

**Table 22:** Parameters with highest number of best prediction results.

Parameter	Description
Algorithm	ANN (*produced best prediction results in 7/11 data sets*)
Input scenario	Input scenario 3 (*produced best prediction results in 8/11 data sets*)
Model	ANN3 (*produced best prediction results in 4/11 data sets*)

**Figure 17:**
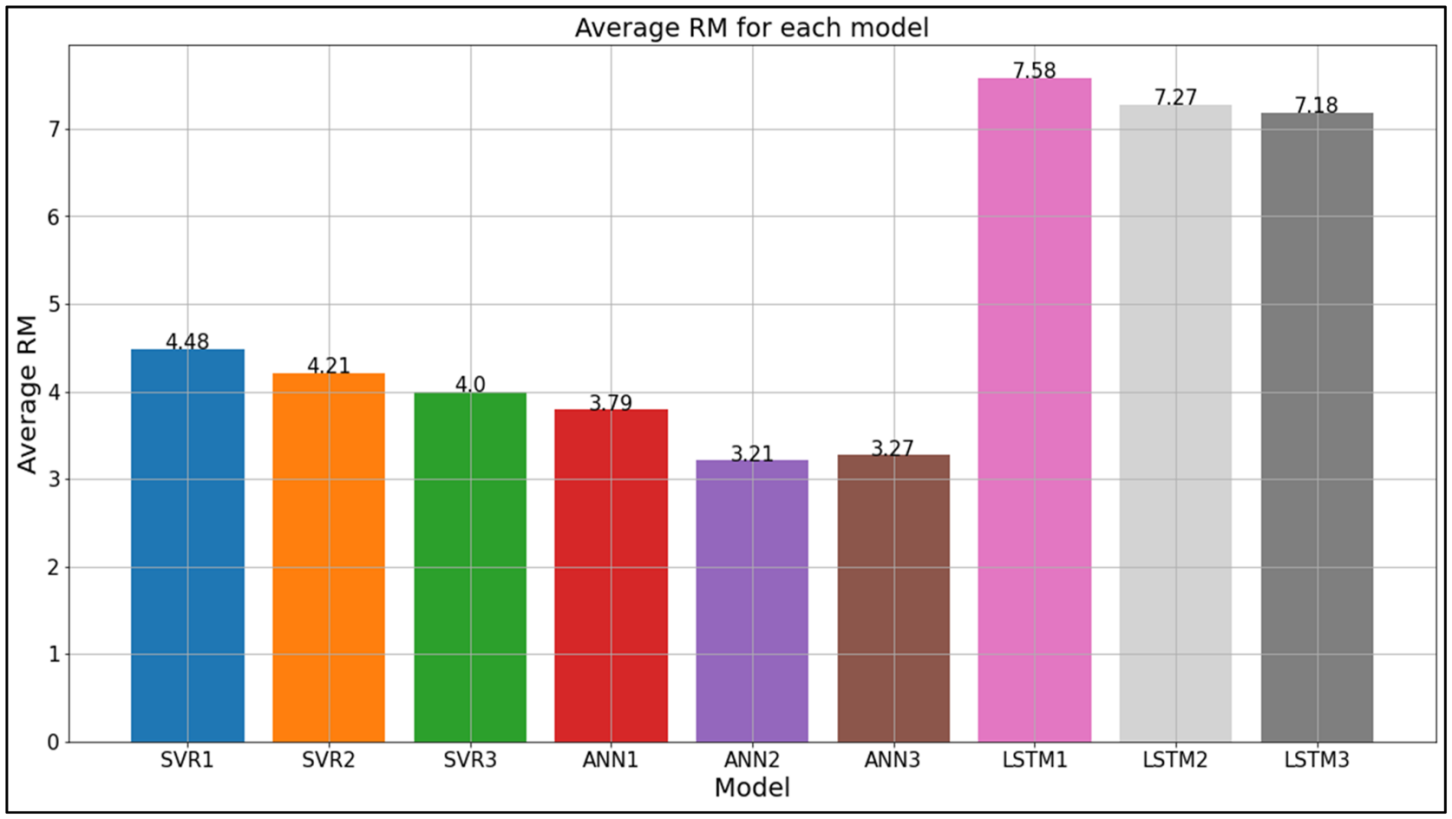
Bar chart of average RM for each model based on all data sets.

Next, the reliability of each model in producing relatively high-accuracy SF predictions based on different data sets is evaluated by calculating and comparing the average of the RM scores obtained by each model for all 11 tested data sets. This evaluation is significant to identify the predictive models that are most robust and most capable of adapting to different data sets which may vary in SF magnitude and behaviour, depending on spatial and temporal factors as well as the heterogeneity of water balance components. Based on Table 23 and Fig. 17, it is determined that ANN2 exhibits the highest average RM with a score of 3.21. This makes ANN2 the most reliable model in predicting SF with a relatively high accuracy for different data sets, in comparison to the other tested models. ANN3 produced the second-best average RM score (average RM = 3.27) which is very close to the ANN2 average RM score, while ANN1 produced the third-best average RM score (average RM = 3.79). Overall, it is found that the top three average RM scores were produced by the ANN models.

**Table 23:** Average RM of each model based on all data sets. Significant values are in bold.

Data set	RM								
	SVR1	SVR2	SVR3	ANN1	ANN2	ANN3	LSTM1	LSTM2	LSTM3
Sungai Johor	6.00	4.00	4.33	3.67	2.00	1.00	8.00	7.00	9.00
Sungai Muda	5.00	3.33	1.67	5.00	2.67	3.33	7.00	8.00	9.00
Sungai Kelantan	4.00	4.33	1.00	3.33	5.67	2.67	8.33	7.33	8.33
Sungai Melaka	3.67	5.00	6.00	1.00	3.33	2.00	7.67	8.33	8.00
Sungai Kepis	5.67	4.67	4.67	8.00	7.00	9.00	2.33	2.67	1.00
Sungai Pahang	6.00	3.33	3.67	5.00	2.00	1.00	9.00	7.33	7.67
Sungai Perak	4.67	4.33	5.33	3.67	1.00	2.00	8.33	8.67	7.00
Sungai Arau	4.00	5.00	6.00	2.00	3.00	1.00	9.00	7.00	8.00
Sungai Selangor	4.67	5.00	5.33	3.00	2.00	1.00	8.67	8.33	7.00
Sungai Dungun	4.00	4.33	4.67	1.00	2.00	5.00	7.00	8.67	8.33
Sungai Klang,	1.67	3.00	1.33	6.00	4.67	8.00	8.00	6.67	5.67
Average RM	4.48	4.21	4.00	3.79	**3.21**	3.27	7.58	7.27	7.18

The best model for SF prediction in the present study is then selected based on the findings with regards to the first evaluation which is the number of times a model produced the best predictive performance for a data set; and the second evaluation which is the reliability of each model in producing SF predictions of relatively high accuracy. For the first evaluation, Table 21 shows that ANN3 was the most accurate SF predictive model for 4 out of the 11 tested data sets, which is more than any of the other tested models. Through the second evaluation, it was found that ANN2 produced the best average RM as shown in Table 23 and Fig. 17, hence indicating that it was the most reliable model in producing relatively high-accuracy SF predictions. Therefore, the two evaluations utilized have proposed different best models, which are ANN2 and ANN3. To make a distinction of the best overall model in the present study, the performances of ANN2 and ANN3 are compared side by side to truly determine the most advantageous SF predictive model. With regards to the first evaluation, it can be seen in Table 21 that there is a clear and significant difference between the performance of ANN2 and ANN3, as ANN2 produced the best SF predictive performance for only 1 out of the 11 tested data sets while ANN3 managed to outperform the other models in 4 out of the 11 tested data sets. Meanwhile, the second evaluation shows that although ANN2 is superior compared to the other models, the difference between the average RMs of ANN2 and ANN3 is very small and negligible as can be seen in Table 23 and Fig. 17. Based on these analyses, ANN3 is selected and proposed as the universal ML model that is capable of predicting SF with high accuracy for rivers within the region of Peninsular Malaysia. Although ANN2 obtained the best average RM score, this model only produced the best predictive performance for 1 out of the 11 tested data sets which is significantly lesser compared to ANN3 which outperformed all the other models for 4 out of the 11 tested data sets, hence why ANN3 was selected as the best model.

Table 21 and Fig. 17 highlight ANN as the most suitable and successful algorithm in the present study, while SVR is the second-best algorithm and LSTM is the poorest performing algorithm. The LSTM predictive performance was significantly poor compared to that of the ANN and SVR algorithms, as the LSTM was only able to outperform ANN and SVR for only one data set while exhibiting the poorest average RMs out of all the algorithms. The poor performance of LSTM in the present study is attributed to the volatility and lack of clear time pattern in the SF data sets, as LSTMs are generally effective in solving problems with clear time patterns. On the other hand, ANN and SVR performed better because they are regression-based methods which appears to be more suited for the current problem of predicting SF in Peninsular Malaysia.

The superiority of the ANN algorithm over the other algorithms in predicting SF may be attributed to the advantages of the ANN algorithm in general. In addition to being able to easily handle large data sets; detect complex non-linear relationships; and easily relate input and output parameters without the need for complex mathematical calculations, the ANN algorithm is also able to learn by itself and produce output or predictions that are not limited to the input provided to it. These advantages appear to have facilitated high-accuracy SF predictive performances by the ANN algorithm, as the ANN algorithm was able to produce the best SF predictive performance for the most data sets (7 out of 11 data sets) compared to the other algorithms. On top of that, it can be seen in Fig. 6 to Fig. 16 that the ANN algorithm predicts the extreme SF values or SF spikes more accurately compared to the other algorithms. Input scenario 3 is found to induce the most success when coupled with the ANN algorithm, as the ANN3 model outperformed all other models in 4 out of the 11 tested data sets while obtaining among the best average RM scores in the present study. This may be because input scenario 3 provides an optimum amount of useful historical SF input that can be used by the ANN algorithm to make accurate SF predictions, hence enabling the ANN3 model to produce highly accurate SF predictions and outperform the other SF predictive models in the present case study.

When compared to existing studies, the findings in the study by Ateeq-ur-Rauf^25^ is agreeable with the findings in the present study, as the ANN algorithm outperforms the SVM algorithm. Additionally, other existing studies also point towards ANN as the superior ML algorithm for SF prediction when compared to other ML algorithms^26–29^. On the contrary, there are also existing studies that contradict the present study’s findings, as they have shown the SVM and LSTM algorithms to perform better in predicting SF compared to the ANN algorithm^6,13,14,16,17,42,43^. This may be due to differences in the experimental setup relating to elements such as input and output parameters; forecast horizons; data set characteristics such as number of data sets and amount of data available for training and testing; study location; magnitude and behaviour of SF in selected river; and ML algorithm hyperparameter setup. In the present study, the SVM algorithm has indeed shown that it is capable of outperforming the ANN algorithm, as it predicted SF better in 3 out of the 11 tested data sets namely the Sungai Muda, Sungai Kelantan, and Sungai Klang data sets. However, the ANN algorithm is superior on an overall scale as it outperformed both the SVM and LSTM algorithms in the remaining 7 tested data sets while also obtaining better average RMs, as shown in Table 21 and Fig. 17. Therefore, it can be summed up that the ANN algorithm is the most accurate and effective ML algorithm for SF prediction when the present study’s experimental setup is applied, which includes a univariate approach that uses lagged daily average SF to predict current daily average SF for 11 different data sets from rivers throughout Peninsular Malaysia. Although the ANN3 model has produced good SF predictive performance in the present study, it can still potentially be improved. Hybridization and usage of optimization algorithms to improve the selection of ML algorithms’ hyperparameters may enhance prediction capability and accuracy. Rainfall data may also be obtained and utilized as an input parameter to improve SF predictive performance, given that rainfall has been shown in existing studies to have a correlation with SF^12,34,61^. These elements are yet to be investigated in the present study; hence they are suggested for future implementations.

## Conclusion

In the present study, daily average SF time series data for 11 different rivers throughout Peninsular Malaysia were collected and utilized for the development of ML models that predict future SF. Three types of ML algorithms were used, namely SVM, ANN, and LSTM. The quantitative analyses show that the ANN3 model, which is based on the ANN algorithm and input scenario 3 (inputs comprising of previous 3 days SF data), represents the best performing model for SF prediction in the present study. ANN3 outperformed all the other tested model in predicting SF for the greatest number of data sets, which is 4 out of the 11 tested data sets. This model also exhibited among the best average RM scores, which indicates that it is highly reliable in producing accurate SF predictions for different data sets which may vary in terms of SF behaviour and magnitude. Additionally, it was found that the algorithm and input scenario that were most effective as model components in predicting SF were ANN and input scenario 3. The ANN algorithm produced the most accurate SF predictions for 7 out of the 11 tested data sets while the usage of input scenario 3 led to the best SF predictions for 8 out of the 11 tested data sets.

In conclusion, the present study set out to address the research gap in which a single ML model capable of accurately predicting SF for multiple different rivers within Peninsular Malaysia is yet to be developed and proposed, as majority of existing studies have focused on the development of SF predictive models based on only one data set or river case study. Therefore, this research gap has been addressed in the present study by developing and testing 99 ML models, based on different established ML algorithms, input scenarios, and SF data sets in Peninsular Malaysia; and proposing the best performing ML model as a universal model that is capable of predicting SF for rivers within the study region. Based on the findings, the present study proposes the ANN3 model as the universal model that is most capable of SF prediction for rivers within Peninsular Malaysia, hence the main objective of the present study is achieved. In hindsight, the findings from the present study are hoped to contribute towards the respective body of knowledge and aid organizations in mitigating the effects of environmental hazards, particularly droughts and floods, through effective and accurate SF predictions using ML models. Further improvement of the ANN3 model for SF prediction in Peninsular Malaysia can be considered as the focus or topic of future studies. Hybridization and utilization of optimization algorithms or more advanced techniques may be used with the ANN3 model to enhance the capability of identifying optimal hyperparameters, resulting in possibly improved accuracy of the model. Rainfall data may also be implemented as an input parameter to improve SF prediction.

## Data Availability

The data that support the findings of this study are available at the Malaysian Department of Irrigation and Drainage.
